# Mechanochemical synthesis of inverse vulcanized polymers

**DOI:** 10.1038/s41467-022-32344-7

**Published:** 2022-08-16

**Authors:** Peiyao Yan, Wei Zhao, Fiona McBride, Diana Cai, Joseph Dale, Veronica Hanna, Tom Hasell

**Affiliations:** 1 Department of Chemistry, University of Liverpool, Crown Street, Liverpool, L69 7ZD UK; 2 Leverhulme Research Centre for Functional Materials Design, Materials Innovation Factory and Department of Chemistry, University of Liverpool, Liverpool, L7 3NY UK; 3 Surface Science Research Centre, University of Liverpool, Liverpool, L69 3BX UK

**Keywords:** Polymer synthesis, Sustainability, Polymers

## Abstract

Inverse vulcanization, a sustainable platform, can transform sulfur, an industrial by-product, into polymers with broad promising applications such as heavy metal capture, electrochemistry and antimicrobials. However, the process usually requires high temperatures (≥159 °C), and the crosslinkers needed to stabilize the sulfur are therefore limited to high-boiling-point monomers only. Here, we report an alternative route for inverse vulcanization—mechanochemical synthesis, with advantages of mild conditions (room temperature), short reaction time (3 h), high atom economy, less H_2_S, and broader monomer range. Successful generation of polymers using crosslinkers ranging from aromatic, aliphatic to volatile, including renewable monomers, demonstrates this method is powerful and versatile. Compared with thermal synthesis, the mechanochemically synthesized products show enhanced mercury capture. The resulting polymers show thermal and light induced recycling. The speed, ease, versatility, safety, and green nature of this process offers a more potential future for inverse vulcanization, and enables further unexpected discoveries.

## Abstract (web summary)

Inverse vulcanization is a process that enables to convert sulfur, a by-product of the petroleum industry, into polymers. Here the authors report a synthetic method of inverse vulcanization via mechanochemical synthesis; compared to thermal routes, a broader range of monomers can be used, and the protocol yields materials with enhanced mercury capture capacity.

## Introduction

Elemental sulfur, as a by-product during the hydrodesulfurization process of crude oil, is extensively produced but has incomplete usage despite its main application in the production of sulfuric acid^1,2^. Hence, there is an urgent need to explore an efficient way to transform this waste into useful materials. Recently, ‘inverse vulcanization’ coined by Pyun and co-workers has given a feasible solution to this issue as >50 wt.% sulfur can be used when a sulfur-containing polymer is formed using this process^3^. The polymers generated by this polymerization route are a new category of material based on a sulfur-sulfur back-bone rather than carbon-carbon back-bone. Consequently, they show many unique properties thanks to their special polymer structures, such as having the highest refractive index among organic materials^4–7^, showing good recycling ability despite having crosslinked structures^8–11^, possessing excellent heavy metal sensitivity arising from the sulfur content^12–15^, and showing antibacterial activity thanks to the inclusion of sulfur^16–18^. Starting from the waste material and turning towards functional applications, inverse vulcanization acts as a sustainable platform of science and technology gives the plastics more sustainable future. Since the first research publication was reported, there has been much related research upon both the underlying chemistry and theoretical, and the potentially applicable directions and applications^19–21^. Majority of renewable resources have been used for production of useful sulfur polymers to be applied in the green areas.

Normally, inverse vulcanized polymers are produced through bulk polymerization at high temperature (≥159 °C), because of the requirements for the cleavage of S–S bonds to enable ring opening and subsequent polymerization of the eight-membered ring (S_8_) by heating. However, there are many accompanying problems in this polymerization process including but not limited to: inhomogeneous polymers obtained caused by less miscible monomers or different reactivity of monomers at high temperature, uncontrollable auto-acceleration^22^, side reactions accompanied by hydrogen abstraction and H_2_S generation^23^, and the limitation of co-monomer choice by boiling point. That is unsafe and hazardous in the operation. In order to apply this new material industrially, alternative easier and safer synthesis methods should be explored. Some attempts have been made regarding to this issue, for example, catalytic synthesis lowered the reaction temperature and unlocked some new monomers, but >100 °C is still needed^24,25^; also, a vapor-phase deposition method which offers an opportunity for more homogeneous reaction and expanded crosslinker range, but it still requires high temperature and harsh conditions^5^.

Here, we have demonstrated that the mechanochemical synthesis, a green method, can be used into the production of inverse vulcanized polymers by using ball milling. Mechanochemistry has a long history from the primaeval mortar and pestle used since the stone age onward^26–29^. Laboratory shaker mills were introduced into chemistry research since the last century and have enabled substantial progress. Up to date, mechanochemistry using ball milling has been extensively exploited in organic synthesis^30,31^, inorganic synthesis^32,33^ and materials synthesis^34,35^. This technique is sourced from mechanical energy and is an environmentally-friendly method which shows advantageous properties including shorter reaction time, homogeneous reaction, high atom economy and so on. It is investigated here that inverse vulcanized polymers can be synthesized by a mechanochemical method. No requirement for heating, fast reaction, solvent-free, reduced hydrogen abstraction, no auto-acceleration, broader monomer options, and more homogeneous reaction regardless of miscibility of sulfur with monomers can all be observed in this polymerization method. According to the obtained results, in addition to the process advantages the method possesses, the polymer materials obtained by the mechanochemical synthesis method show many unexpected and interesting properties compared with the normal thermally synthesized products, which are discussed in detail below. A notable and surprising finding was that the fallen iron filings from the steel milling balls are able to chemically react with the polymers to form thermally stable inorganic substances rather than being only physically dispersed in the polymers.

Briefly, in this work we report an alternative synthetic method of inverse vulcanization—mechanochemical synthesis, which allows a broader range of monomer options and brings unique properties to relevant materials, and is a potentially greener, safer, and more efficient process compared the current thermal routes is presented in this work.

## Results

### Synthesis and characterizations of mechanochemically synthesized inverse vulcanized polymers

**Fig. 1:**
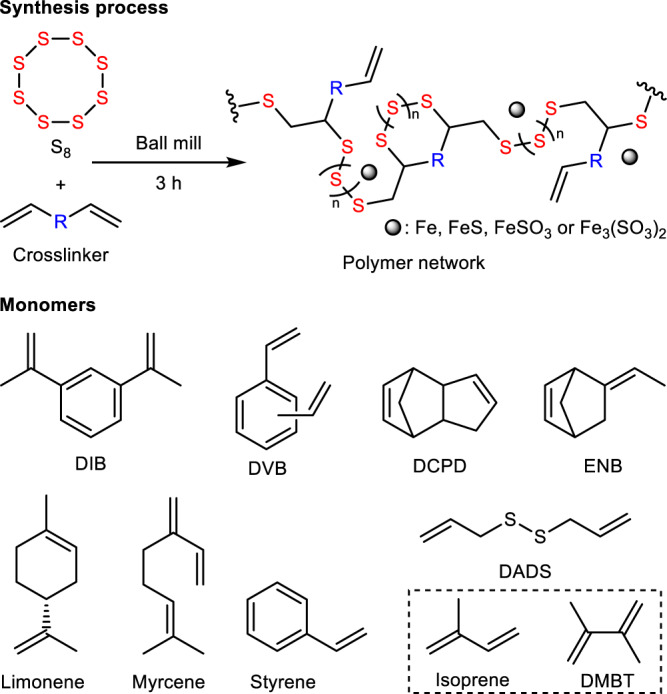
Reaction scheme of mechanochemical synthesis of inverse vulcanized polymers and monomers used. The top panel shows the synthesis process of mechanochemically synthesized inverse vulcanized polymers, and bottom panel shows all monomers used in the reactions and low-boiling point monomers are marked with dotted box.

We synthesized ten polymers using a ball mill, starting from different crosslinkers ranging from aromatic, aliphatic to volatile monomers including 1,3-diisopropenylbenzene (DIB), dicyclopentadiene (DCPD), divinylbenzene (DVB), 5-ethylidene-2-norbornene (ENB), limonene, myrcene, dially disulfide (DADS), styrene, 2,3-Dimethyl-1,3-butadiene (DMBT) and isoprene (Fig. 1). The synthetic polymers were named as MS(S-monomer) respectively. In addition, 8 polymers derived from the same monomers, omitting DMBT and isoprene which are unable to be used at high temperature, were synthesized using the conventional thermal synthesis method to act as control group, and were named as TS(S-monomer) respectively. The experimental procedures of the materials synthesis can be found in the ‘Methods’ section. MS(S-DIB) and MS(S-Styrene) were used as model reactions to optimize the reaction procedure, as DIB is a well-known monomer, which can chemically stabilize polymeric sulfur, and styrene is a monomer with lower stabilizing efficiency for sulfur because of the lower number of reactive bonds relative to the molecular mass, and linear resulting structure. As differential scanning calorimetry (DSC) curves in Supplementary Fig. 2 show, the obtained polymer MS(S-DIB) shows a clear glass-rubber transition even after only 1-h of reaction, and a clear increase of glass transition temperature (*T*_*g*_) from −11 °C to −1 °C with the reaction time increase from 1 h to 3 h, suggesting that DIB can react with sulfur quickly to form sulfur-polymer by ball milling, and further crosslinking reaction occurs during extended reaction time. However, MS(S-Styrene) still has an obvious melt peak of unreacted crystalline sulfur even after 2-h reaction, and there is only a slight glass-rubber transition apparent. After reacting for 3 h, there is no unreacted crystalline sulfur remaining in polymerization system, and the product has a very clear glass-rubber transition. That indicates that styrene needs longer time to fully react with sulfur compared with DIB, and is able to form a sulfur polymer after 3-h reaction.

Hence, based on the results of optimization experiments, all other polymers were synthesized for 3 h in order to fully convert the S_8_. To be clear here, all the characterizations of the products were carried out once the polymers were formed without any further treatment unless there is a special illustration. The DSC curves of all the polymers (Supplementary Fig. 3) show that every polymer has a clear *T*_*g*_ and almost all polymers have no unreacted crystalline sulfur remaining except a slight sulfur melt peak in MS(S-Myrcene) after 3-h polymerization. It’s worth noting that the volatile monomer isoprene (b.p. 34 °C) and low-boiling point monomer DMBT (b.p. 68 °C) were proven to successfully react with sulfur to form sulfur polymer by mechanochemical synthesis method here, which definitely cannot be realized through the conventional thermal synthesis process. Comparing the *T*_*g*_ of mechanochemically synthesized products with that of thermally synthesized products (Supplementary Fig. 4), it is interestingly found that some of the mechanochemically synthesized products have much lower *T*_*g*_ than the relevant thermally synthesized products synthesized from the crosslinkers including DIB, DCPD, DVB, ENB and DADS, which would form crosslinked network normally, but other mechanochemically synthesized products formed from monomers including myrcene, limonene and styrene, show slightly higher *T*_*g*_ than the relevant thermally synthesized products which are tend to be formed as linear polymer normally. From the powder x-ray diffraction (PXRD) curves of the mechanochemically synthesized products in Supplementary Fig. 5, we can see that there is a trace of crystalline sulfur persisting in some of the polymers although it is not apparent from the second heating cycle in DSC curves. The unreacted sulfur can be removed by Soxhlet extraction in acetone as evidenced by the absence of peaks in PXRD patterns (Supplementary Fig. 6a) of the mechanochemically synthesized products after Soxhlet extraction, but it needs to be mentioned that further reaction could occur during heating resulting from the dynamic disulfide bonds, since the DSC results (Supplementary Fig. 6b) which show an increase of *T*_*g*_ of all the polymers after Soxhlet extraction. Hence, all the characterizations of mechanochemically synthesized polymers were done directly after 3-h reaction in the ball mill to investigate their true chemical and physical properties and applications. According to solubility evaluation results in Supplementary Fig. 7, all the mechanochemically synthesized polymers were demonstrated to be insoluble in tetrahydrofuran (THF), chloroform, and acetone, which is notable. As Supplementary Fig. 8 shows, TS(S-Myrcene), TS(S-Limonene), and TS(S-Styrene), which either exhibit linear or branched rather than fully crosslinked structures, are fully soluble in THF and chloroform and partly soluble in acetone. Other thermally synthesized polymers, which are more highly crosslinked, are insoluble or only partly soluble in those three solvents. Theoretically, higher crosslinking degree will give higher *T*_*g*_ and higher solvent resistance to the polymer. Here one question arises, why should some of the mechanochemically synthesized polymers possess lower *T*_*g*_ than the thermally synthesized polymers, but show higher solvent resistance? Elemental analysis results in Supplementary Table 2 shows that most of obtained polymers contain high sulfur content but there are changes on the ratios of C:H and C:S after polymerization. The most suspected way which can cause elemental ratio change is the side reaction—H_2_S gas generation in thermal synthesis, which can cause C:H ratio and C:S ratio increase as elements S and H are lost. However, the C:H ratio and C:S ratio here both decreased after mechanochemical synthesis. So, another question comes up—how this happened? Both questions suggest there might be something different from mechanochemical synthesis method to the normal thermal synthesis method in mechanism.

### Investigating the properties of mechanochemical reaction of inverse vulcanized polymers

**Fig. 2:**
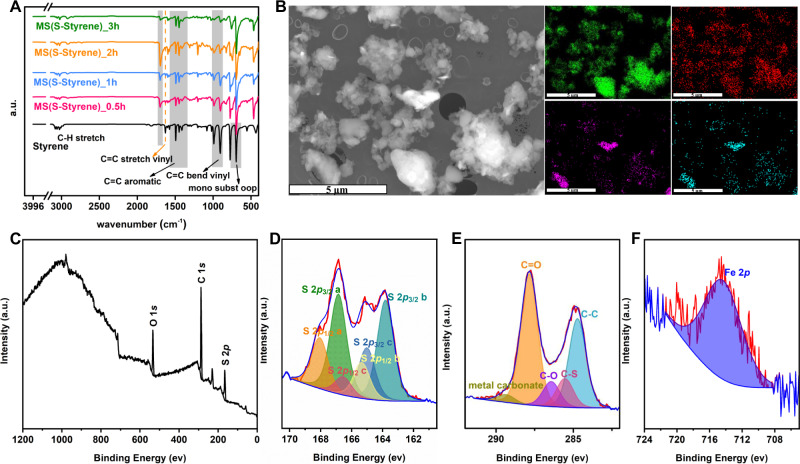
Structure and morphology characterizations. **A** Synthesis of MS(S-Styrene) monitored by using FT-IR. **B** SEM and EDS images (green is elemental S, red is elemental C, purple is elemental Fe, and blue is elemental Gr) of product MS(S-DIB) with same size bar (5 µm). **C**–**F** Show XPS spectra of MS(S-Styrene); wide scan, and corresponding S 2*p*, C 1* s*, and Fe 2*p* spectra with associated curve fits.

In order to investigate these questions and better understand the nature of the chemical reaction in the ball mill, styrene was chosen as model monomer to monitor the reaction as it theoretically has the least reactive activation point. Fourier transform infrared spectroscopy (FT-IR), hydrogen nuclear magnetic resonance spectroscopy (^1^H NMR), and X-ray photoelectron spectroscopy (XPS) were used to monitor the chemical environment changes as a function of reaction time. From FT-IR curves in Fig. 2A, it is observed that the peak at ~1626 cm^−1^ belonging to the C=C stretch and peaks at ~989 cm^−1^ and ~906 cm^−1^ belonging to C=C bend both disappeared with the increase of the reaction time, which suggests that vinyl groups in styrene were consumed to form polymer. Meanwhile, there is no change of the peaks belonging to the benzene ring, suggesting that there is no reaction occurring between the benzene group and sulfur, but there is a new peak at ~1701 cm^−1^ appearing after reaction, which is attributed to a C=O group. The same results can be observed from the FT-IR spectra of TS(S-Styrene) (Supplementary Fig. 9). In addition, DSC curve (Supplementary Fig. 10) of the control experiment MS(S-Toluene) suggests that there is no reaction between sulfur with saturated hydrocarbon. Due to insoluble property of the MS(S-Styrene), it is difficult to obtain the structure information of the produced polymer using solution NMR, but it is clear that the monomer styrene is fully consumed after 3-h reaction from the ^1^H NMR spectra (Supplementary Figs. 11, 12) of monomer and products, resulting from the disappearance of the peaks belonging to hydrogen protons of styrene. In addition, energy dispersive spectroscopy (EDS) was used to detect the elements existing in the polymers. It is surprisingly found that there is Fe present in addition to the elements S and C in the polymer from the EDS images of MS(S-DIB) in Fig. 2B. That indicates that there are some iron extracted from steel balls trapped in the polymers, and there is a trace of Cr associated with Fe existing in the polymer, which is normally used in steel production. Moreover, Supplementary Fig. 13 indicates that Fe was not uniformly dispersed in the polymer. In this case, anchoring of the polymeric products to Fe was considered to be the probable reason for the insolubility and unexplained elemental analysis results of mechanochemically synthesized products. The presence of the Fe impurities prohibits the use of solid-state NMR. Hence, XPS was used to investigate bonding information of the mechanochemically synthesized polymers by recording the C 1 *s*, S 2*p*, Fe 2*p* and O 1 *s* regions (Fig. 2C). As Supplementary Fig. 14 shows, clearly there are more S and C chemical environments observed from MS(S-DIB) or MS(S-Styrene) than that of TS(S-DIB) and TS(S-Styrene). Figure 2F clearly shows the presence of Fe in the polymer MS(S-Styrene), and Fig. 2D, E give the detailed connectivity information of polymer MS(S-Styrene). XPS S 2*p* peak in Fig. 2D is fitted by three components: neutral S (163-165.2 eV) and cationic S^+^ (165.3–166.7 eV) and oxidized SO^3-^ (166.8-168.4 eV)^36^. The S 2*p* spectrum was curve fitted, indicating the presence of four sulfur chemical bonding environments; S–S, C–S, S–O and S with Fe at a binding energy (BE) of 163.8 eV, 165.0 eV, 165.4 eV/166.6 eV and 166.9 eV/168.1 eV, respectively^5,37,38^. Furthermore, the bonding assignments from the C 1 *s* data, Fig. 2E, show five carbon bonding environments; C–C, C–S, C–O, C=O and metal carbonate at a BE of 284.7 eV, 285.5 eV, 286.4 eV, 287.9 eV, 289.3 eV respectively^36,39^. That means that sulfur has reacted with the organic co-monomers and chemically connected with carbon to form C–S bonds, and there are bonds of metal sulfonate and metal carbonate formed in the meantime. In contrast, XPS S 2*p* and C 1 *s* regions (and accompanying survey scan) of TS(S-Styrene) (Supplementary Fig. 14d) show no Fe present, carbon and sulfur were only observed in only the following bonding environments; S–S (163.9 eV), C–S (165.0 eV in S 2*p* spectrum and 285.49 eV in C 1 *s* spectrum), and C–C (284.7 eV in C 1 *s* spectrum). Hence, it is further demonstrated that new covalent bonds of C–S were formed in mechanochemically synthesized polymer, and Fe not only disperses in the polymer physically but chemically connects with the polymer chains. Moreover, XPS spectra of MS(S-DIB) and TS(S-DIB) show similar results with that of MS(S-Styrene) and TS(S-Styrene) (Supplementary Fig. 14a, b). Therefore, all the above proof illustrates that sulfur did react with C=C bonds in the monomers initiated by mechanical energy to successfully form the sulfur polymers, and iron from the steel balls reacted with the polymer to form some inorganic proportion accompanying the polymer formation rather than only being ground iron dispersed among polymer particles, which is surprising and interesting.

Solution inductively coupled plasma optical emission spectrometry (ICP-OES) was used to analyze Fe content of the polymers. As Supplementary Table 3 shows, there are ranging from 8 wt.% to 20 wt.% Fe in the polymers but there is only 0.77 wt.% Fe in the control sample--ball milled pure elemental sulfur. Furthermore, thermogravimetric analysis (TGA) of mechanochemically synthesized polymers in N_2_ shown in Supplementary Fig. 15 illustrates that there still are some residue percent from polymers at 1000 °C, but only around 1.4% residue of control sample (ball milled sulfur) was obtained. It is revealed that Fe only reacts with polymer chains during polymer formation but does not react with pure sulfur. This result is supported by some publications which have proved that polysulfides can oxidize zero valent metals such as mercury and that polysulfides are more reactive than elemental sulfur in such processes^40–42^. Now, we can understand that why the ratios of C:H and C:S decrease after polymerization in EA results. That is because that percentage of metal cannot be detected using EA technique, but the total percentages of elements were calculated basing on total polymers’ mass, which causes decrease of carbon percentage. In addition, unreacted monomers were washed out by acetone before the EA characterization, which further decreases the carbon percentage. Hence, the detected carbon percent is lower than the calculated value so that the ratios of C:H and C:S are lower than the calculated values. That also indicates that there may be no/less H_2_S formation during reaction as there is no decrease of sulfur percentage. Theoretically, the organic material all can be burned off in the air. However, the synthesized materials in this case still have Fe oxide remaining after being burned, which can be proved by the EDS images of burned MS(S-DIB) in Supplementary Fig. 16, there is only elements Fe, Ge or some C which cannot be confirmed since the background is carbon film, but no sulfur remaining. Hence, it is clear that an inorganic fraction formed and iron from the steel balls cause the questions discussed above. Fe reacted with the polymer chain and causes structure change to the polymer resulting in insolubility, where Fe acts as a kind of crosslinker to link the polymer chain into the network. While, the FT-IR curves of some of the polymers in Supplementary Figs. 17–25 show that partial reaction of C=C of monomer containing 2 or more vinyl groups occured, which causes those mechanochemically synthesized polymers to have lower *T*_*g*_ than the thermally synthesized polymers. Whereas, some monomers which potentially form highly linear structured polymer, like styrene which has the least C=C so that it has high sulfur rank and high opportunity for Fe to attend the reaction to form high content of inorganic fractions, resulted in the formed polymer having higher *T*_*g*_ than the thermally synthesized polymer. Furthermore, a ceramic ball mill (made with zirconia) was used to see whether iron is necessary for reaction. As the results show in Supplementary Figs. 26–33 and Supplementary Table 5, inverse vulcanized polymers can also be formed by using a ceramic ball mill. That suggests that mechanical force is the drive force of the reaction rather than metal existence.

**Fig. 3:**
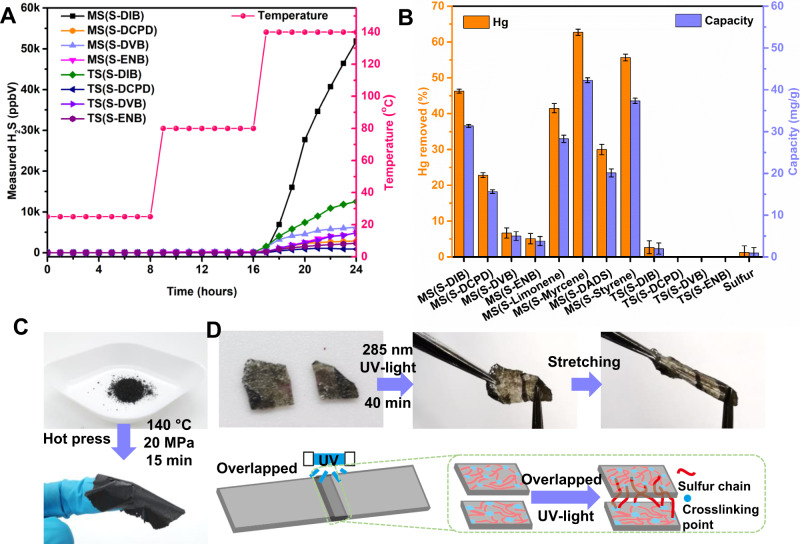
H_2_S measurements and potential application explorations. **A** H_2_S measurements on selected mechanochemically synthesized and thermally synthesized polymers with ramping temperature at room temperature, 80 °C and 140 °C. **B** The percentage mercury removed from 138 ppm HgCl_2_ solution after 24 h exposure to each of the materials listed, and the capacity of mercury removing of each of the materials listed. (Here, error bar is standard deviation of the three replicate test results.) **C** Polymer MS(S-DIB) as an example to show the polymer film is able to be made from polymer powder. **D** Polymer MS(S-ENB) film as an example to show the UV-induced self-healing ability of the mechanochemically synthesized polymer.

In addition, H_2_S gas measurements were done to evaluate the hydrogen adsorption in both of mechanochemical synthesis and thermal synthesis polymerization. As it is hard to monitor the reaction during the reaction due to the practical limitations of the equipment, the products were used to analyze H_2_S generation tests. Polymers TS(S-DIB), TS(S-DCPD), TS(S-DVB) and TS(S-ENB) were chosen as the control samples to compare with the mechanochemically synthesized polymers from the same crosslinker respectively, as they all are solid state at room temperature. From Fig. 3A, it can be seen that H_2_S is only generated under heating. Slight H_2_S concentration can be detected from some of mechanochemically synthesized products once the temperature reaches 80 °C, but a significant H_2_S concentration can be detected when the temperature is 140 °C no matter whether mechanochemically synthesized polymers or thermally synthesized polymers. mechanochemically synthesized products show quicker H_2_S generation rate than thermally synthesized products as they are smaller particles with higher surface area and shorter diffusion pathways. Even through the thermally synthesized polymers are fully crosslinked polymers, there was significant H_2_S generation at high temperature. This suggests that H_2_S gas is likely released during the thermal synthesis process thanks to the high reaction temperature (normally >140 °C). Instead, it can be considered as that there is likely no/less H_2_S gas generated during the mechanochemical synthesis process due to absence of heating. Additionally, the H_2_S concentrations released from mechanochemically synthesized polymers are all higher than that from thermally synthesized polymers (the data for every polymer can be found in Supplementary Figs. 34–41). In theory, less gas generated from the products suggests hydrogen abstraction may have already occurred during the synthesis process as the same monomers and ratio were used in synthesis. Therefore, for the reaction itself, there should be less or no H_2_S generated during the mechanochemical synthesis process, in comparison to the thermal synthesis process.

### Exploration of the potential applications of mechanochemically synthesized inverse vulcanized polymers

Moreover, scanning electron microscopy (SEM) images of the mechanochemically synthesized polymers (panels a and b in each of Supplementary Figs. 42–49) show all the polymers are relatively small size particles. Accordingly, the obtained polymer powders were considered as a sorbent in water remediation. Hence, mercury (Hg) uptake tests were carried out using all the mechanochemically synthesized polymers with the control samples of solid-state thermally synthesized polymers and pure sulfur. The experimental procedures of Hg uptake can be found in the ‘Methods’ section. As Fig. 3B show, all the mechanochemically synthesized polymers have higher Hg capture efficiency than the thermal synthesized polymers as well as pure sulfur. The maximum 62.72 ± 0.85% Hg was removed from actual 138 ppm HgCl_2_ solution by 20 mg polymer MS(S-Myrcene) in 24 h and the highest capacity of 42.28 ± 0.57 mg/g was obtained, which is higher than normally non-porous sulfur polymers and even higher than some kinds of porous polymers reported^22,43,44^, such as salt templated sulfur polymer which has the capacity of 2.27 mg/g^43^. Even more, 99.07 ± 0.06% Hg was removed from actual 9.54 ppm HgCl_2_ solution by 20 mg polymer MS(S-Myrcene) after 24 h. Deriving from renewable monomers and industrial by-product sulfur, combined with the enhanced mercury removal efficiency, the mechanochemically synthesized materials let inverse vulcanized polymers be in a wider sustainable stage. Even more, the mechanochemically synthesized materials have a high potential for industrial use, as they can be easily, safely, and effectively produced, and as-made materials can be directly used for heavy metal sorbents, only requiring filtration before use. Additionally, the mechanochemically synthesized polymer powders can be processed further by hot pressing into polymer thin film, as shown in Fig. 3C, despite their crosslinked structures. That will give the mechanochemically synthesized materials wider applications. Supplementary Figs. 50, 51 and Supplementary Table 6 show that mechanochemically synthesized MS(S-DIB) is stronger and stiffer than the thermally synthesized TS(S-DIB) (the tensile strength and Young’s modulus of the former one is >10-fold higher than that of the later one), while the later one is more stretching than the former one with much higher breaking strain. As the polymers contain S–S bonds that can be broken and reformed, the polymer thin film was expected to have potential for healing. It is known that dynamic reaction between the disulfide bonds can be introduced by heat^45^ and also ultraviolet (UV) light^46^. Thermally induced healing properties of inverse vulcanized polymers has been widely investigated^8,9^, but we demonstrate that the inverse vulcanized polymer is able to be self-healed under UV light irradiation. Figure 3D indicates that two pieces of MS(S-ENB) films were healed together after radiation using 285 nm UV light for 40 min. The healed film shows a good elastic property as the Supplementary Movie 1 shows, where the film can be stretched and then the deformation can be recovered automatically once the force is released.

## Discussion

In summary, it has been demonstrated that inverse vulcanized polymers can be synthesized using a mechanochemical method. In the conventional thermal synthesis of inverse vulcanized polymers, the choice of potential crosslinkers is constrained to those that are miscible with molten sulfur as well as having sufficiently high-boiling points^47^. The mechanochemical route removes these constraints. It was proven that iron from steel balls can react with the sulfur polymer to form inorganic sections anchoring the polymers. The obtained polymers were demonstrated to show many modified properties such as high solvent resistance and high mercury uptake efficiency and capacity. The synthetic materials also show good processing ability which might be used to broaden applications not limited to UV-induced self-healing. Compared with the normal thermal synthesis, mechanochemical synthesis method of inverse vulcanization is able to give broader monomer choice and promising product range simply starting from the by-product, sulfur, to wider applications.

Although the mechanochemical synthesis method of inverse vulcanization has been demonstrated to possess such many advantages, we could not ignore that there are some potential disadvantages of this method. We can see from the photographs of the products that all the polymers show relatively dark color probably caused by iron from the ball mill, which might will limit their applications in optical materials. However, other potential promising applications which do not have requirements on materials’ color, such as cathode materials used for Li-S battery, could be explored by designing specific polymers’ structure combined with adding conductive fillers during synthesis in an easy way. It was shown that the mechanochemically synthesized polymers have higher mechanical properties than those synthesized thermally, but the strength is still limited compared with commercial plastics, therefore, it is worth to investigate how to improve the mechanical properties of mechanochemically synthesized inverse vulcanized polymers in future research, either by designing new monomers or blends, or by adding fillers. Generally, despite the materials and applications which have been demonstrated in this work, further monomers and potential applications of the mechanochemically synthesized inverse vulcanized polymers call for further exploration.

## Methods

### Synthesis of polymers MS(S-monomer)

The mechanochemical synthesis of inverse vulcanized polymers were carried out using weight ratio of 1:1 between the elemental sulfur with the crosslinker. Around 400 mg reaction mixture was added into a stainless-steel milling jar (10 ml) with adding 5 stainless-steel ball (diameter, 2 mm) (The photo of the equipment can be found in Supplementary Fig. 1). Milling frequency was 30 Hz, and reaction time was 3 h. After reaction, the obtained products were filtered by washing with acetone. Mechanochemically synthesized polymers are named as MS(S-crosslinker), and crosslinkers are shown in Fig. 1.

For control synthesis experiments using ceramic ball mill, zirconia jar (10 ml) and zirconia ball (diameter, 2 mm) were used. Number of balls, frequency used, and reaction time were consistent with steel milling process.

### Synthesis of polymers TS(S-monomer)

In order to compare the properties of mechanochemically synthesized products with thermal synthesized polymers, a batch of thermally synthesized polymers TS(S-crosslinker) were produced using same crosslinkers. The reaction temperature and reaction time for each polymer can be found in the Supplementary Table 1.

### Characterizations of synthesized polymers

Nuclear magnetic resonance spectroscopy (NMR), fourier transform infrared spectroscopy (FT-IR), x-ray photoelectron (XPS), differential scanning calorimetry (DSC), thermogravimetric analysis (TGA), powder x-ray diffraction (PXRD), scanning electron microscopy (SEM) and energy dispersive spectroscopy (EDS), inductively coupled plasma optical emission spectrometry (ICP-OES), selected ion flow tube mass spectrometer (SIFT-MS), and tensile test were conducted in this study. All the facilities and conditions of each characterization can be found in the supplementary information file.

### Solubility evaluation

The solubility of obtained polymers was evaluated by using three different solvents (THF, chloroform and acetone). The concentrations of polymers were prepared as around 3 mg/ml and the mass of each polymer was recorded. After the solvent was added in, the solution was stirred for 24 h by using an agitator. The photographs of the solubility of each polymer in each solvent have been recorded.

### Solution ICP-OES sample preparation

In order to detect the concentration of elemental ion in the generated polymers, solution ICP-OES was performed for each polymer. As all polymers are insoluble, they were digested in nitric acid by using microwave. The procedure of the digestion is: ∼50 mg of each polymer was added in 8 ml nitric acid (67–69%, trace metal analysis grade), then was digested by using a standard HDPE procedure in the Perkin Elmer Microwave. The digestion solution was diluted into 52 ml solution by using deionised (DI) water. Then 10 ml of each sample was filtered out using 0.2 μm nylon filter and was submitted for solution ICP-OES analysis.

### Mercury uptake procedure

1. A theoretical 100 ppm mercury chloride (HgCl_2_) solution (250 ml) was made up from a stock solution of HgCl_2_ and DI water. 10 ml of each solution was added into a series of glass vials along with 20 mg of each polymer. All the polymers were ground by using a pestle and mortar before exposed in to HgCl_2_ solutions. The vials were then placed on an agitator for stirring 24 h at room temperature. The test solutions were filtered into clean polypropylene centrifuge tubes using 0.2 µm nylon filter with a ten-fold dilution and 1 mL nitric acid (10%) for stabilization. A water blank and a theoretical 100 ppm control sample were prepared in same procedure. All test samples were submitted for an analysis using the same calibration method on the ICP-OES. The actual concentration of HgCl_2_, the uptake percentage and the capacity of polymers can be analyzed according to the ICP-OES results.
2. A theoretical 10 ppm mercury chloride (HgCl_2_) solution (50 ml) was made up from a stock solution of HgCl_2_ and DI water. 10 ml of the solution was added into a glass vial along with 20 mg of polymer MS(S-Myrcene). The polymer was ground by using a pestle and mortar before exposed in to HgCl_2_ solutions. The vial was then placed on an agitator for stirring 24 h at room temperature. The test solution was filtered into clean polypropylene centrifuge tubes using 0.2 µm nylon filter and 1 mL nitric acid (10%) for stabilization. A water blank and a theoretical 10 ppm control sample were prepared in same procedure. All test samples were submitted for an analysis using the same calibration method on the ICP-OES. The actual concentration of HgCl_2_ and the uptake percentage of polymer can be analyzed according to the ICP-OES results.

### Making thin film using hot-press and UV-induced self-healing evaluation

Mechanochemically synthesized polymer powders were able to be processed into thin film by using hot press purchased from Zhengzhou CY Scientific Instrument CO., LTD. Polymers MS(S-DIB) and MS(S-ENB) were hot pressed at 140 °C and under 20 MPa for 15 min. After cooling down to room temperature, the polymer film was demoulded. MS(S-ENB) thin film was used into UV-induced self-healing ability investigation. Two pieces of thin films were overlapped for around 1 mm width gap and were covered by two glass slides and then were irradiated by 285 nm UV light for 40 min (Analytikjena 4 Watt UVLS-24 EL Series UV Lamp). Before and after healing, the states of polymer films were recorded by photographs and video. The temperature of the polymer surface was monitored every 5 min (by infrared thermometer), and the temperature range remained between 23.5-25.5 °C during the 40-min UV irradiation

## Supplementary information

Supplementary information

Peer Review File

Description of Additional Supplementary Files

Supplementary Movie 1

## Data Availability

Data supporting the findings in this study is fully available within the main content and the supplementary information file.
