# Supplementary material for: Mechanochemical synthesis of inverse vulcanized polymers
Source: Nat Commun. 2022 Aug 16;13:4824. doi: 10.1038/s41467-022-32344-7 (PMC9381570; doi:10.1038/s41467-022-32344-7)
Supplement: Supplementary file 1 — Supplementary information [file 41467_2022_32344_MOESM1_ESM.pdf]

Supplementary Information

Mechanochemical synthesis of inverse vulcanized polymers

Peiyao Yan‡*[1], Wei Zhao‡[1,2], Fiona McBride[3], Diana Cai[1], Joseph Dale[1], Veronica Hanna[1], Tom Hasell*[1]

[1]Department of Chemistry, University of Liverpool, Crown Street, Liverpool L69 7ZD, UK; [2]Leverhulme Research Centre for Functional Materials Design, Materials Innovation Factory and Department of Chemistry, University of Liverpool, Liverpool L7 3NY, UK; [3]Surface Science Research Centre, University of Liverpool, Liverpool L69 3BX, UK.

*Email: Peiyao.Yan@liverpool.ac.uk; t0m@liverpool.ac.uk.

‡=these authors contributed equally; *=corresponding authors

Supplementary Methods

Material Sulfur (S_8, ≥99.5 %), 1,3-diisopropenylbenzene (DIB), dicyclopentadiene (DCPD), divinylbenzene (DVB), 5-ethylidene-2-norbornene (ENB), limonene, myrcene, dially disulfide (DADS), styrene, isoprene, tetrahydrofuran (THF), chloroform, acetone, toluene, dimethylacetamide (DMAc), dichloromethane (DCM), and chloroform-d ($CDCl_3$) were purchased from Sigma Aldrich and used as received without further purification.

Characterizations **1. Nuclear magnetic resonance spectroscopy (NMR):** Solution NMR of unreacted monomers was recorded using a Bruker Advance DRX (400 MHz) spectrometer. $CDCl_3$ was used as solvent, and insoluble fractions were filtered by using a 2 µm filter. **2. Fourier transform infrared spectroscopy (FT-IR):** FT-IR was carried out using Vertex 70 Fourier Transform Infrared Spectrometer between 400 cm^{-1} to 4000 cm^{-1} for 32 scans under resolution of 4 cm^{-1}. FT-IR spectrum of solid polymers were measured by using ATR attachment with diamond, and the liquid cell with 1mm spacer was used for hazardous monomers. Data was analysed using the software OPUS. **3. X-ray photoelectron (XPS):** XPS Analysis was performed using a Kratos Axis SUPRA XPS fitted with a monochromated Al kα X-ray source (1486.7 eV), a spherical sector analyser and 3 multichannel resistive plate, 128 channel delay line detectors. All data was recorded at 150 W and a spot size of 700 x 300 µm. Survey scans were recorded at a pass energy of 160 eV, step size 1.0 eV and high-resolution scans recorded at a pass energy of 20 eV, step size 0.1 eV. Electronic charge neutralization was achieved using a magnetic immersion lens. Filament current = 0.27 A, charge balance = 3.3 V, filament bias = 3.8 V. All sample data was recorded at a pressure below 10^{-8} Torr and a room temperature of 294 K. Data was analysed using CasaXPS v2.3.24PR1.0 and the spectra were calibrated with C 1s peak at 284.8 eV. **4. Differential scanning calorimetry (DSC):** DSC was carried out using Q2000 DSC (TA instruments). The method with heat/cool/heat for three cycles at a heating/cooling rate of 10 °C/min and with ranging from -80 ºC to 150 ºC was performed. The second heating curves were recorded and analysed, and glass transition temperatures of polymers were analysed using TA software. **5. Thermogravimetric analysis (TGA):** The thermal stability of polymer was conducted using a TA Instruments 550 under nitrogen from room temperature to 1000 ºC at a heating rate of 10 ºC min^{-1}. 10 mg-20 mg of samples were used for testing. **6. Powder x-ray Diffraction (PXRD):** PXRD measurements were performed on a PANalytical X'Pert PRO MPD, using in high transmission mode with K_α focusing mirror and PIXCEL 1D detector with Cu X-ray source over 2θ range from 1º to 55º over 30 minutes. **7. Scanning electron microscopy (SEM) and energy dispersive spectroscopy (EDS):** The micrographs and EDS spectra were acquired when the microscope was operated in SEM mode by using two different equipment (Hitachi S-4800 and Tescan S8000G). When the Hitachi S-4800 was used, all samples were placed on the silica surface and

were coated using chromium before the test. When the Tescan S8000G was used, the specimens were prepared on TEM carbon grids. **8. Inductively coupled plasma optical emission spectrometry (ICP-OES):** Metal (Hg^{2+}, Fe, and Cr) concentrations were analysed by using an Agilent 5110 ICP-OES spectrometer, equipped with an autosampler. **9. Selected ion flow tube mass spectrometer (SIFT-MS):** Hydrogen sulphide analysis was conducted on a Selected ion flow tube mass spectrometer (SIFT-MS) operating with O_2^+, H_2O^+, and NO_2^+ reagent ions. 100 mg polymer samples were prepared in vials, with 10 micro litre headspace samples removed at hourly intervals and analysed assessing the occurrence of H_2S^+ ions (registering at a mass of 35 Daltons). **10. Tensile test:** Tensile testing were carried out using a universal testing machine (SHIMADZU EZTest) with crosshead rate of 5 mm/min. The testing temperature was fixed at 21±1 °C using air conditioner. Rectangle testing samples (L (30 mm) *W (5 mm) *T (0.1 mm)) were used. Samples were cut from polymer thin films, which were made by using hot press. The experimental procedure of making polymer films is illustrated in the results and discussion section. The average values with error bar of all mechanical data were obtained after 5 times tests for every sample.

Experimental procedures

1. Synthesis of polymers MS(S-monomer). The photo of the equipment is shown in Supplementary Fig. 1. **2. Synthesis of polymers TS(S-monomer).** The reaction temperature and reaction time followed the reported procedures.[1, 2, 3, 4, 5] The synthesis procedures of all polymers are shown in the supplementary Table 1.

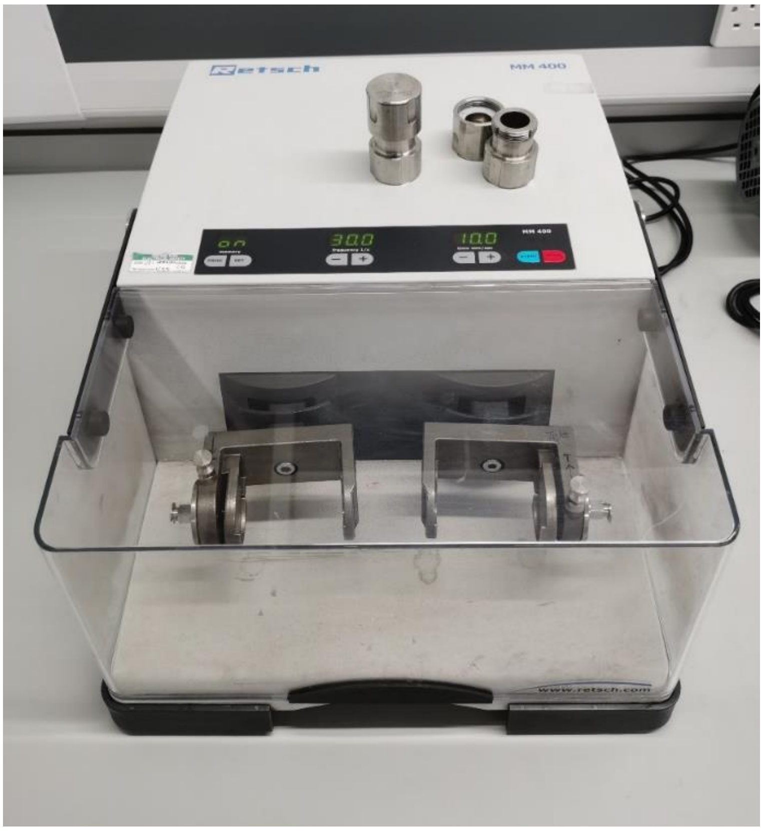

Supplementary Fig. 1. The photograph of the ball mill which was used in the polymer synthesis.

Supplementary Table 1. Polymerization conditions of polymers TS(S-monomer).

Sample	Reaction temperature/reaction time	Curing temperature/curing time
TS(S-DIB)	160 °C/40 mins	15 hrs/140 °C
TS(S-DCPD)	160 °C /20 mins	15 hrs/140 °C
TS(S-DVB)	160 °C /1 hrs	15 hrs/140 °C
TS(S-ENB)	135 °C /35 mins	15 hrs/140 °C
TS(S-Limonene)	160 °C /2 hrs	15 hrs/140 °C
TS(S-Myrcene)	160 °C /1 hr and 10mins	15 hrs/140 °C
TS(S-DADS)	160 °C /30 mins	15 hrs/140 °C
TS(S-Styrene)	130 °C /4 hrs	15 hrs/130 °C

Supplementary discussion

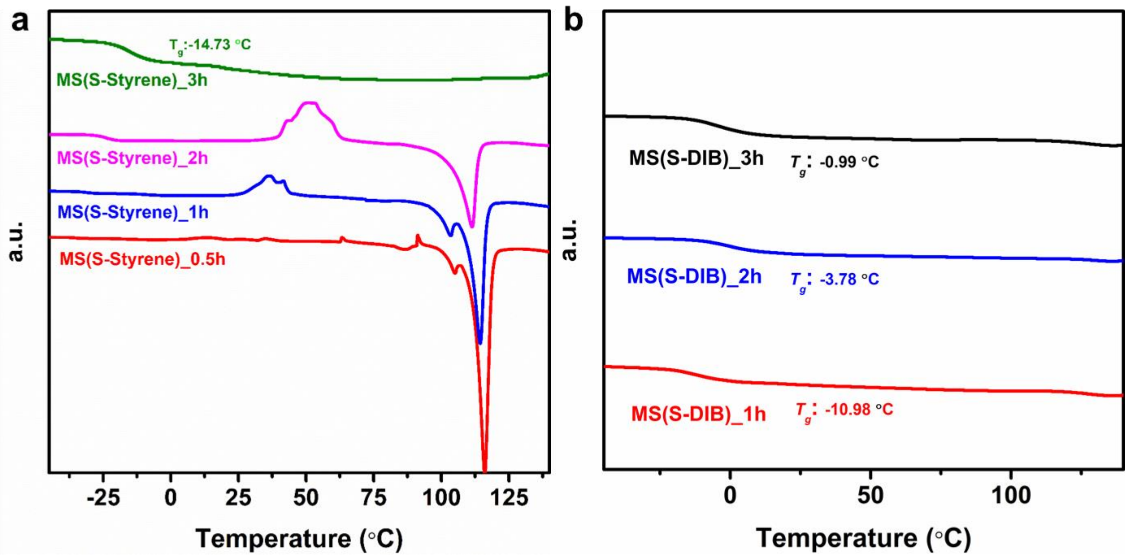

Supplementary Fig. 2. Reaction monitor using DSC of a) MS(S-Styrene) and b) MS(S-DIB).

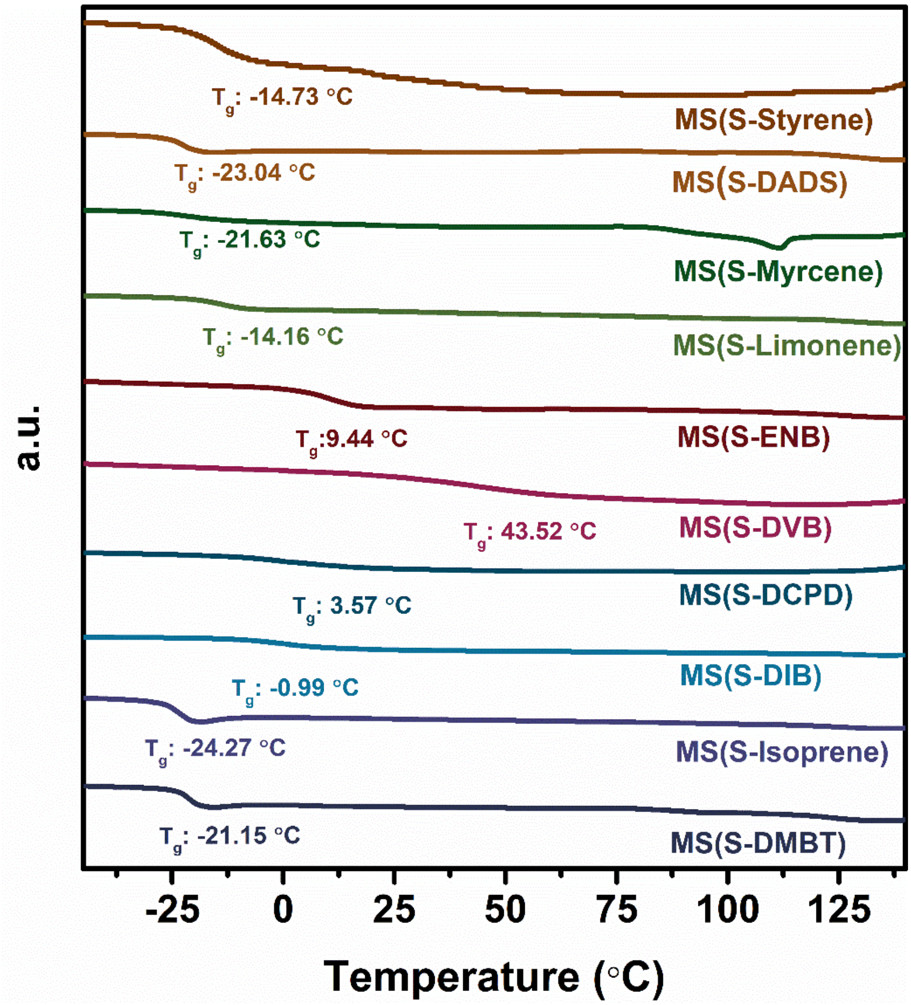

Supplementary Fig. 3. DSC curves of polymers MS(S-monomer).

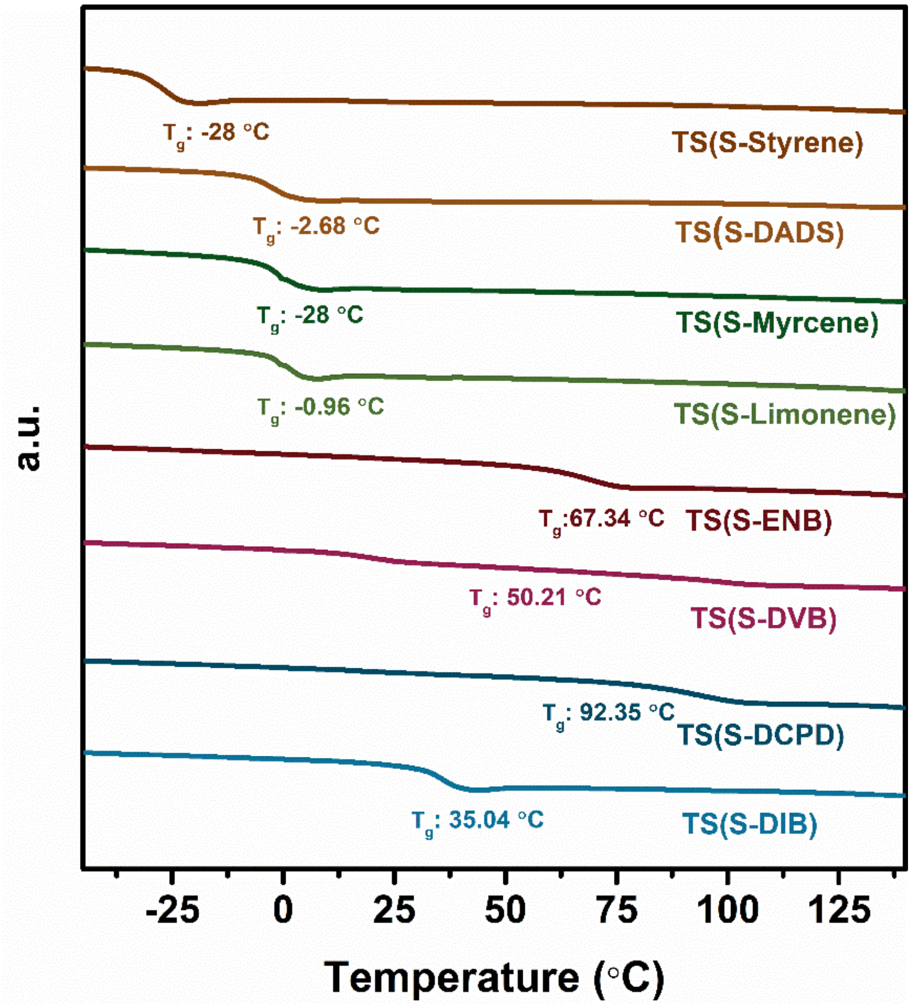

Supplementary Fig. 4. DSC curves of polymers TS(S-monomer).

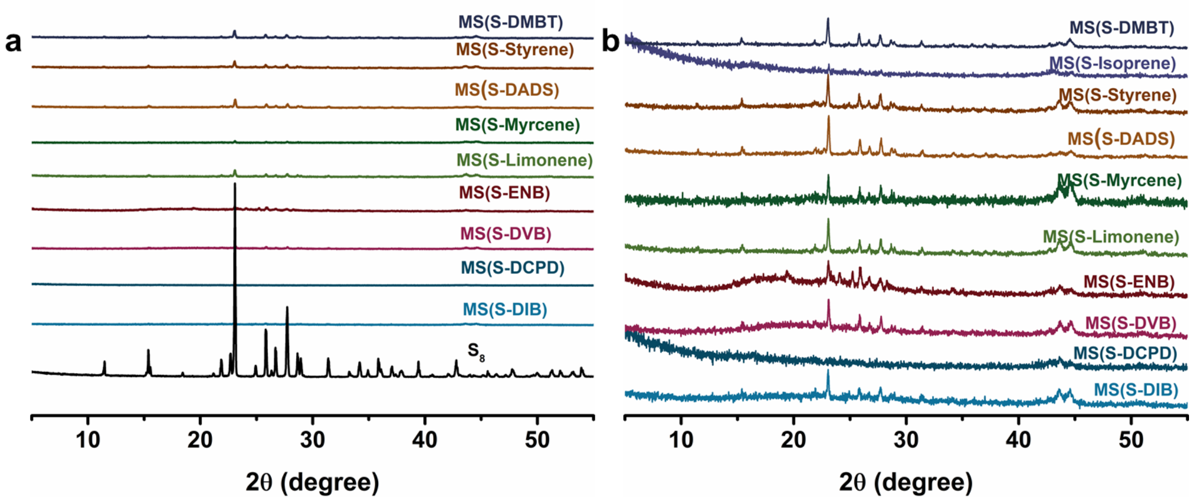

Supplementary Fig. 5. a) PXRD curves of the MS products compared with pure sulfur, and b) zoom of the PXRD curves of the mechanochemically synthesized products removing the PXRD curve of pure sulfur.

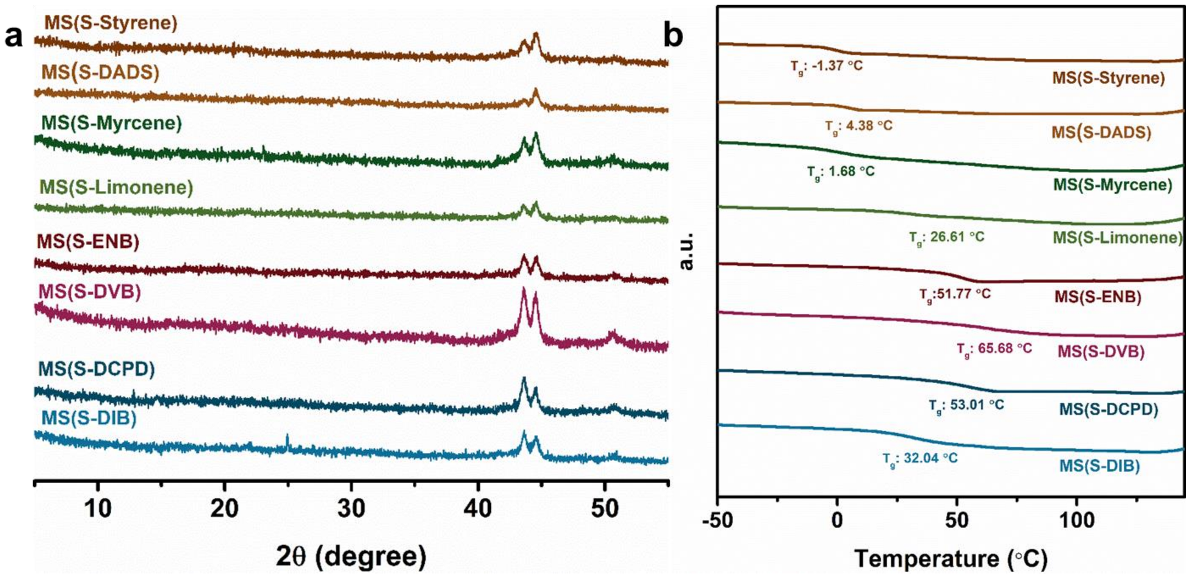

Supplementary Fig. 6. a) PXRD curves and b) DSC curves of the mechanochemically synthesized products after Soxhlet extraction.

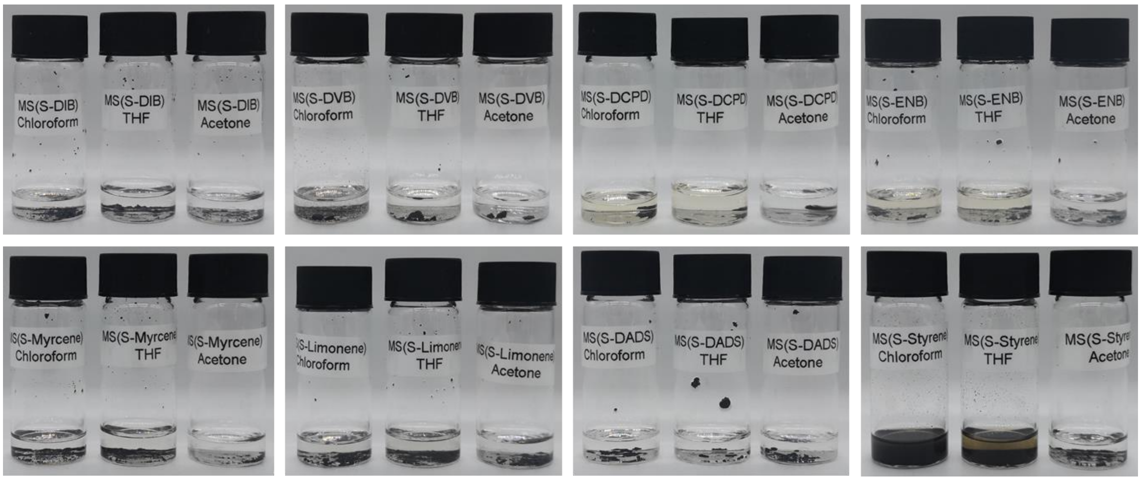

Supplementary Fig. 7. Photographs of solubility evaluation of the mechanochemically synthesized products using solvents THF, chloroform and acetone.

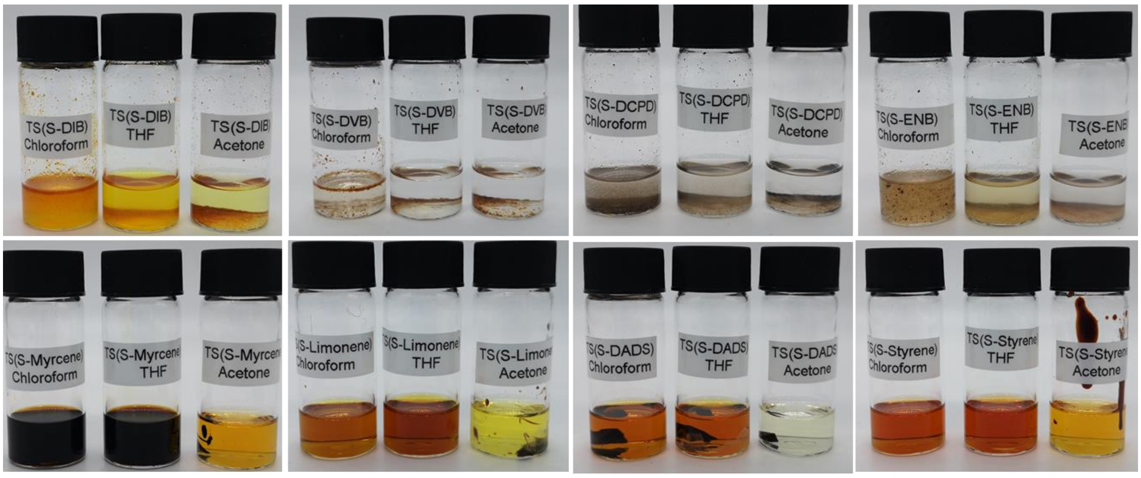

Supplementary Fig. 8. Photographs of solubility evaluation of the thermally synthesized products using solvents THF, chloroform and acetone.

Supplementary Table 2. Elemental analysis (EA) results of mechanochemically synthesized products.

	Calc %					Analysis 1 (without any further treatment)				
Sample Ref	%C	%H	%S	C:H	C:S	%C	%H	%S	C:H	C:S
MS(S-DIB)	45.50	4.50	50.00	10.11	0.91	13.40	2.10	62.30	6.38	0.22
MS(S-DCPD)	45.50	4.50	50.00	10.11	0.91	22.40	3.00	42.50	7.47	0.53
MS(S-DVB)	46.20	3.80	50.00	12.16	0.92	14.93	2.05	55.00	7.28	0.27
MS(S-ENB)	45.00	5.00	50.00	9.00	0.90	4.80	1.40	58.00	3.43	0.08
MS(S-Limonene)	43.50	6.50	50.00	6.69	0.87	5.40	1.40	65.00	3.86	0.08
MS(S-Myrcene)	43.50	6.50	50.00	6.69	0.87	11.00	1.80	45.50	6.11	0.24
MS(S-DADS)	31.60	4.40	64.00	7.18	0.49	1.60	1.18	85.00	1.36	0.02
MS(S-Styrene)	46.00	4.00	50.00	11.50	0.92	9.90	1.30	36.70	7.62	0.27
MS(S-Isoprene)	44.10	5.90	50.00	7.47	0.88	10.09	1.60	70.80	6.31	0.14
MS(S-DMBT)	43.87	6.14	50.00	7.14	0.88	10.68	1.95	70.03	5.48	0.15
BM(Sulfur)[1]	0	0	100	0	0	0	1.2	99.00	0	0

[1]BM(Sulfur) refers to ball milled elemental sulfur

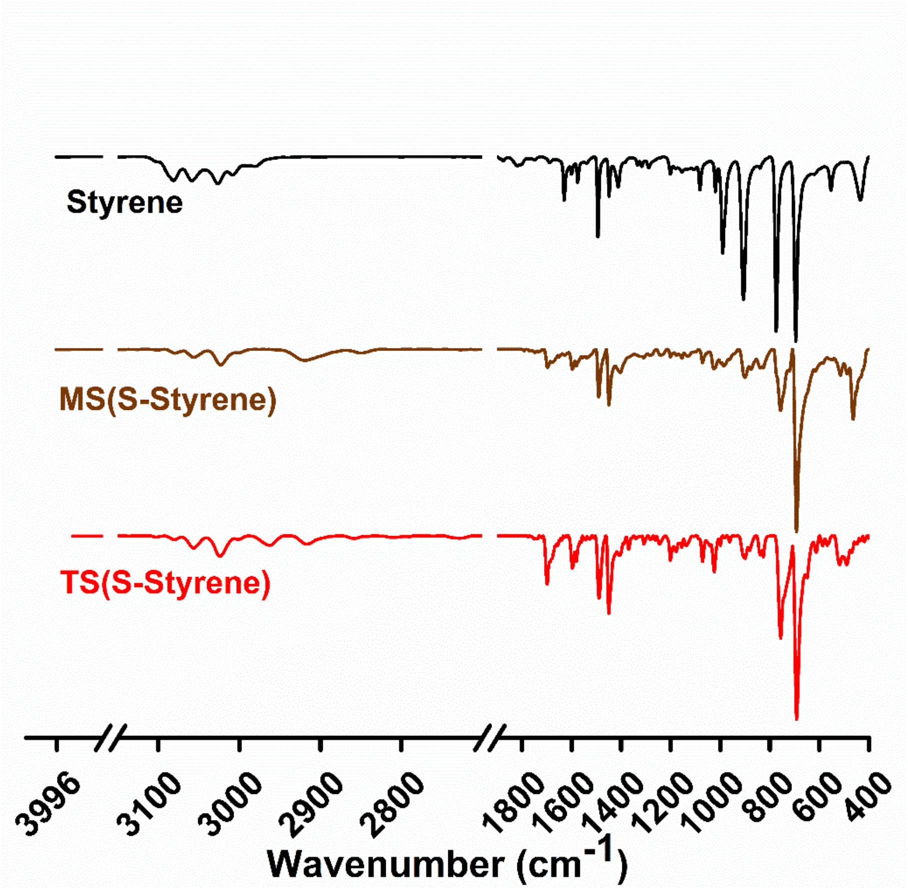

Supplementary Fig. 9. FT-IR curves of monomer styrene, polymer MS(S-Styrene) and TS(S-Styrene).

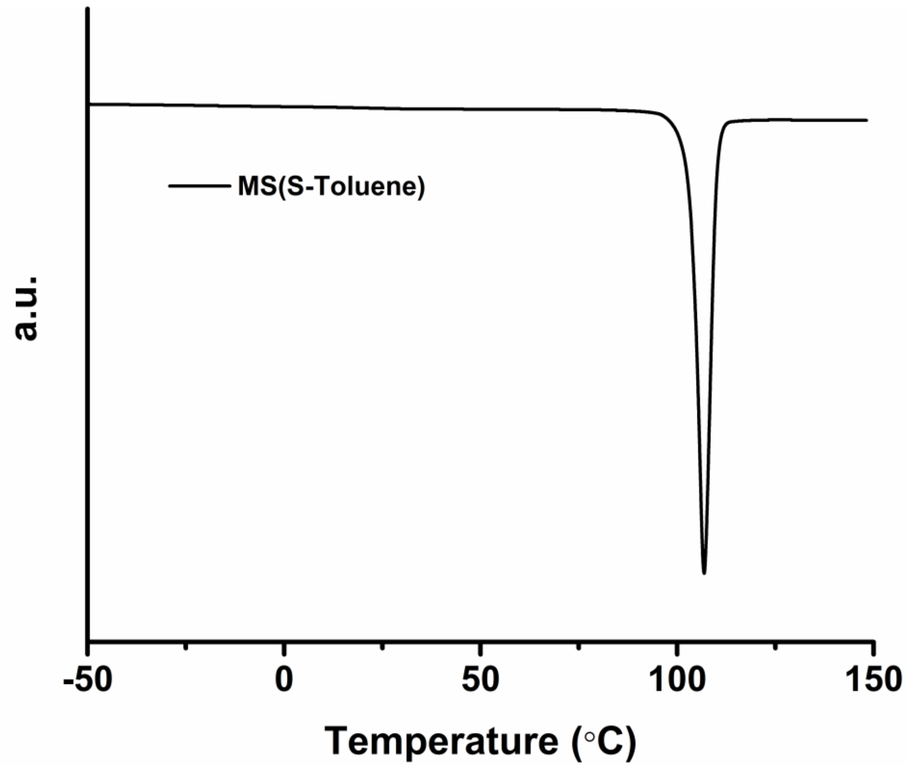

Supplementary Fig. 10. DSC curve of MS(S-Toluene).

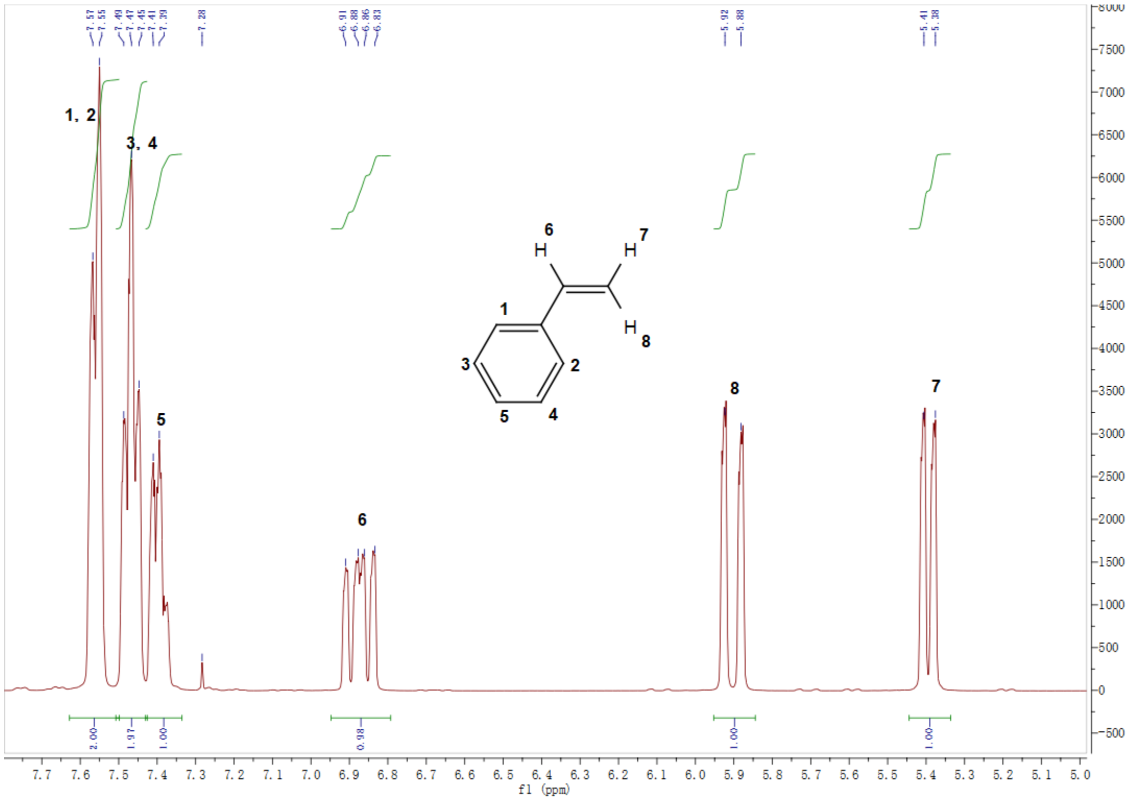

Supplementary Fig. 11. ^{1}H NMR spectrum of monomer styrene, conducted at room temperature (~22 ºC). 400 MHz spectrometer and solvent CDCl3 were used.

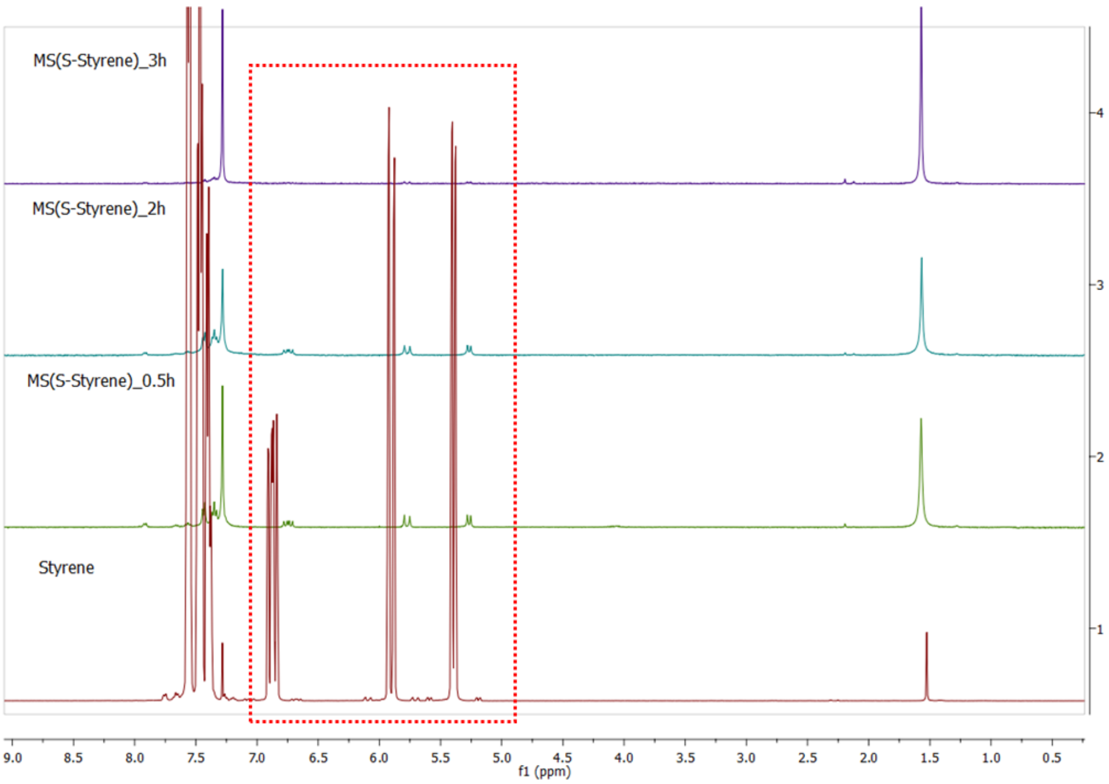

Supplementary Fig. 12. ^{1}H NMR spectra of MS(S-Styrene) monitored with reaction time at 0.5h, 2h and 3h, conducted at room temperature (~22 °C). 400 MHz spectrometer and solvent CDCl3 were used.

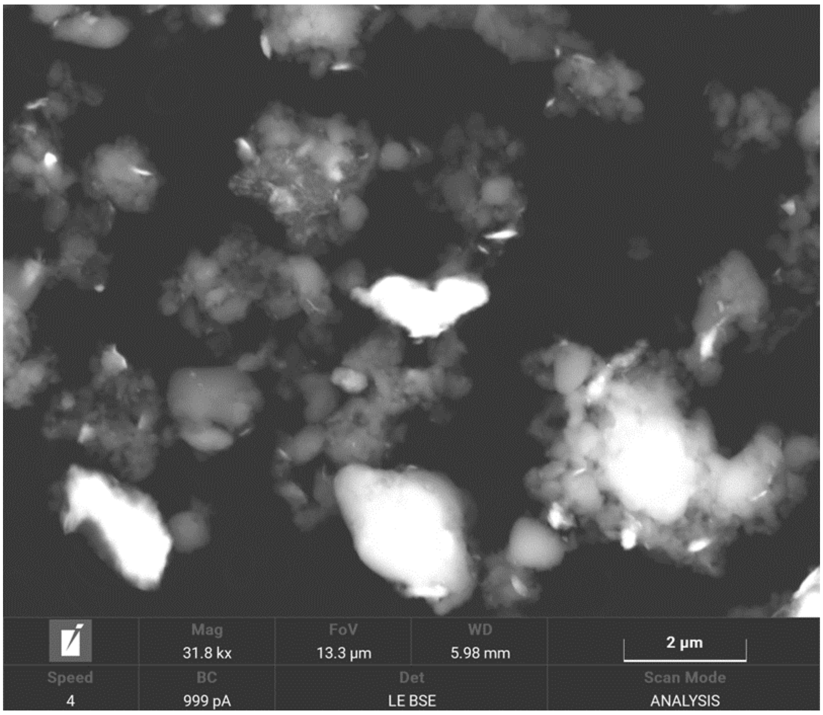

Supplementary Fig. 13. SEM image of MS(S-DIB). Bright part is full of elemental Fe.

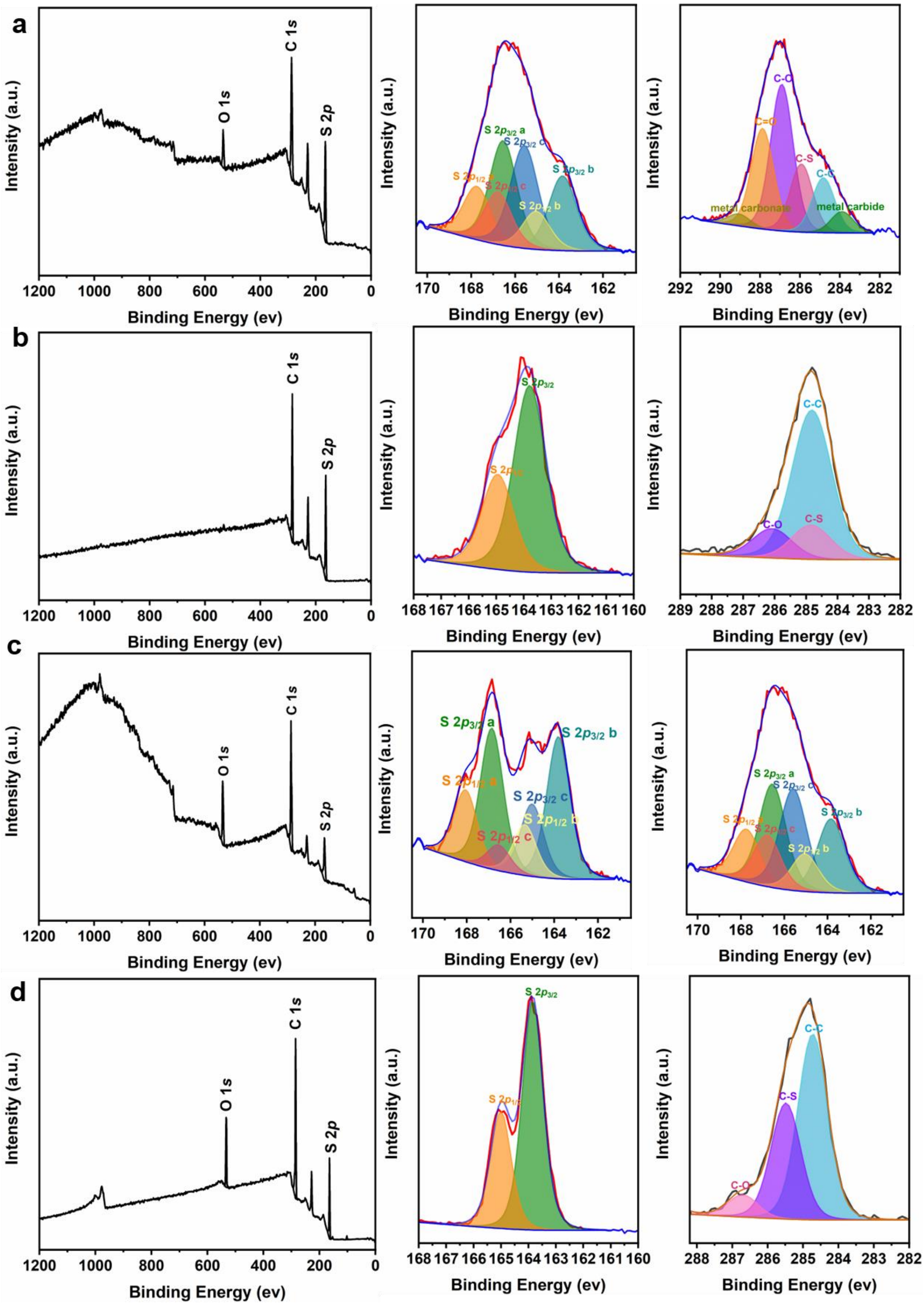

Supplementary Fig. 14. XPS survey scan, S 2*p* and C 1*s* spectra (with associated curve fits) of a) MS(S-DIB) b) TS(S-DIB) c) MS(S-Styrene) and d) TS(S-Styrene).

Supplementary Table 3. Fe content of polymers analysed from solution ICP results.

Sample Ref	Fe (%)	Cr (%)
MS(S-DIB)	13.65±0.19	3.60±0.12
MS(S-DCPD)	20.35±0.31	5.08±0.02
MS(S-DVB)	17.93±0.23	4.53±0.00
MS(S-ENB)	12.84±0.03	3.08±0.02
MS(S-Limonene)	11.45±0.15	2.86±0.01
MS(S-Myrcene)	12.84±0.13	3.21±0.00
MS(S-DADS)	8.82±0.11	2.20±0.00
MS(S-Styrene)	8.15±0.09	2.02±0.00
BM(Sulfur)[1]	0.77±0.00	0.19±0.00

[1]BM(Sulfur) refers to ball milled elemental sulfur

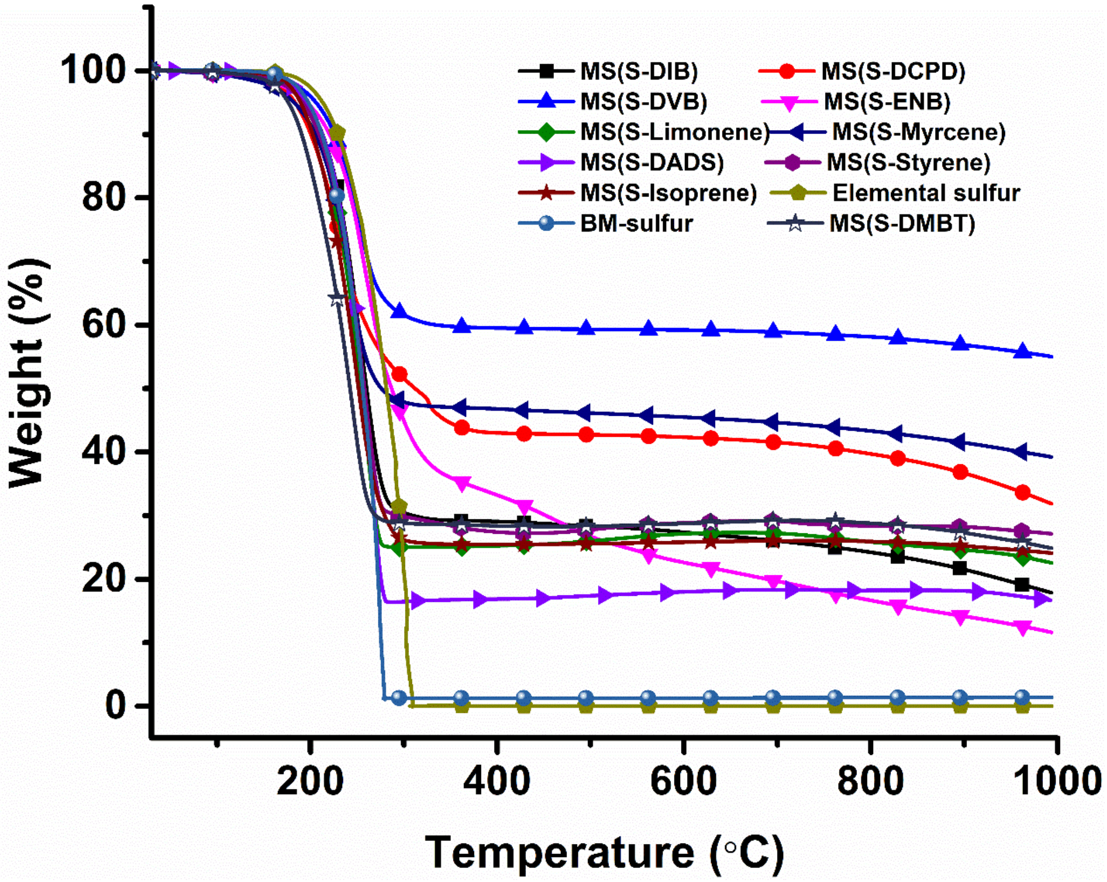

Supplementary Fig. 15. TGA curves of mechanochemically synthesized polymers in the N_2.

Supplementary Table 4. A summary of values of Fe percent from solution ICP, missing percent from EA and residue percent from TGA in N_2.

Sample	Fe percent from solution ICP (%)	Missing percent from EA (%)	Residue percent from TGA in N_2 (%)
MS(S-DIB)	13.65±0.19	22.20	18.0
MS(S-DCPD)	20.35±0.31	32.10	32.0
MS(S-DVB)	17.93±0.23	28.02	55.1
MS(S-ENB)	12.84±0.03	35.80	11.7
MS(S-Limonene)	11.45±0.15	28.20	22.6
MS(S-Myrcene)	12.84±0.13	41.70	39.4
MS(S-DADS)	8.82±0.11	12.22	16.7
MS(S-Styrene)	8.15±0.09	52.10	27.2
BM(Sulfur)	0.77±0.00	1.00	1.4

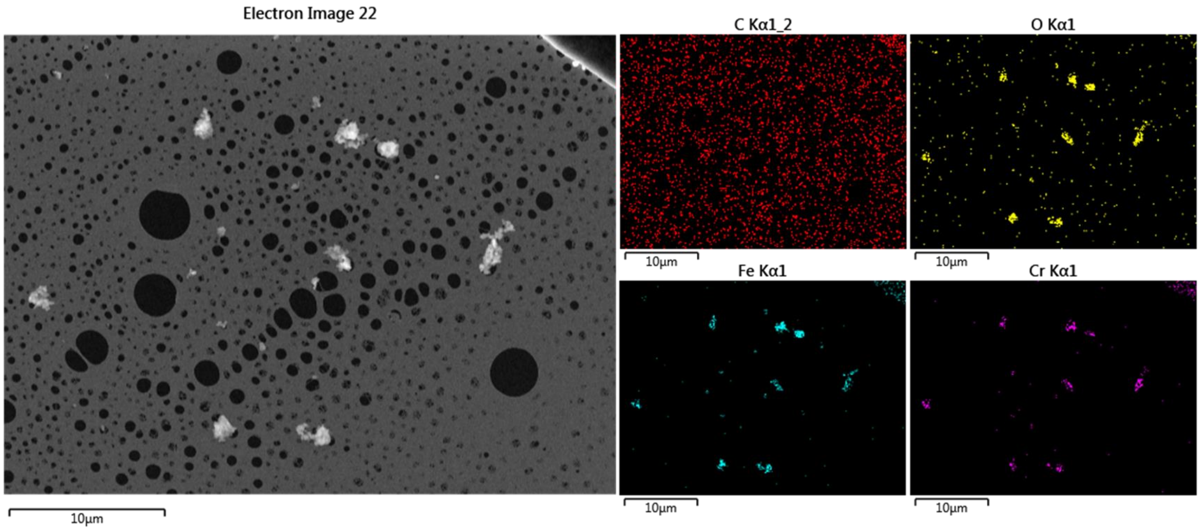

Supplementary Fig. 16. SEM image and EDS images of MS(S-DIB) after burning.

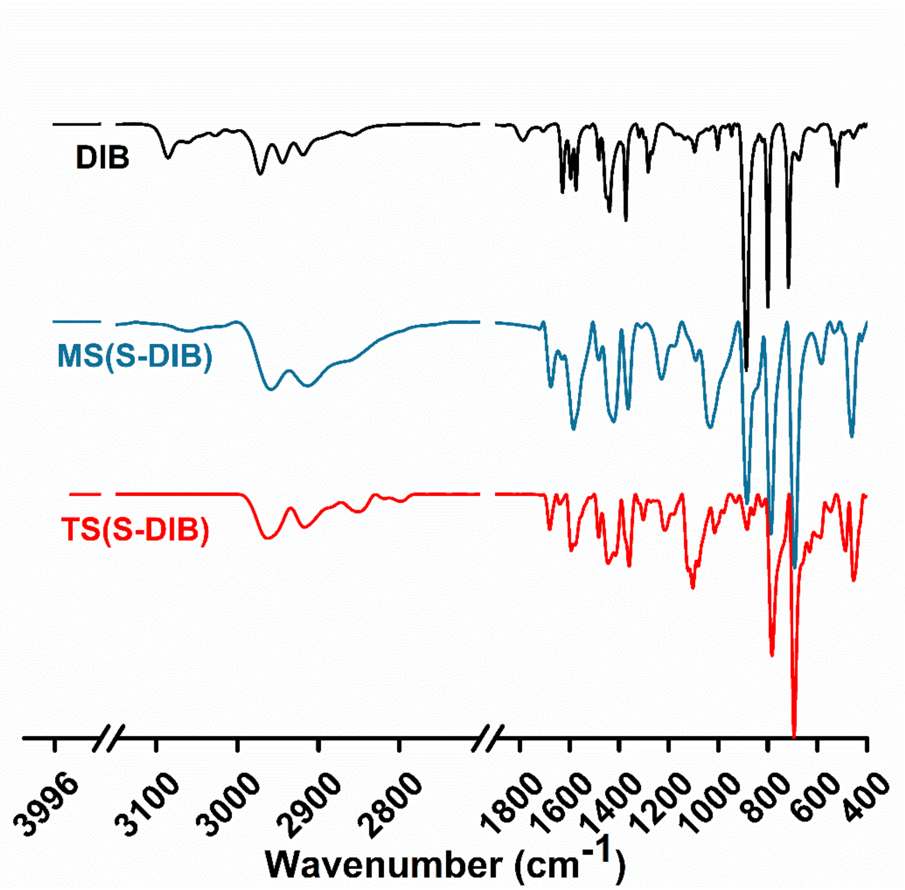

Supplementary Fig. 17. FT-IR curves of monomer DIB, polymer MS(S-DIB) and TS(S-DIB).

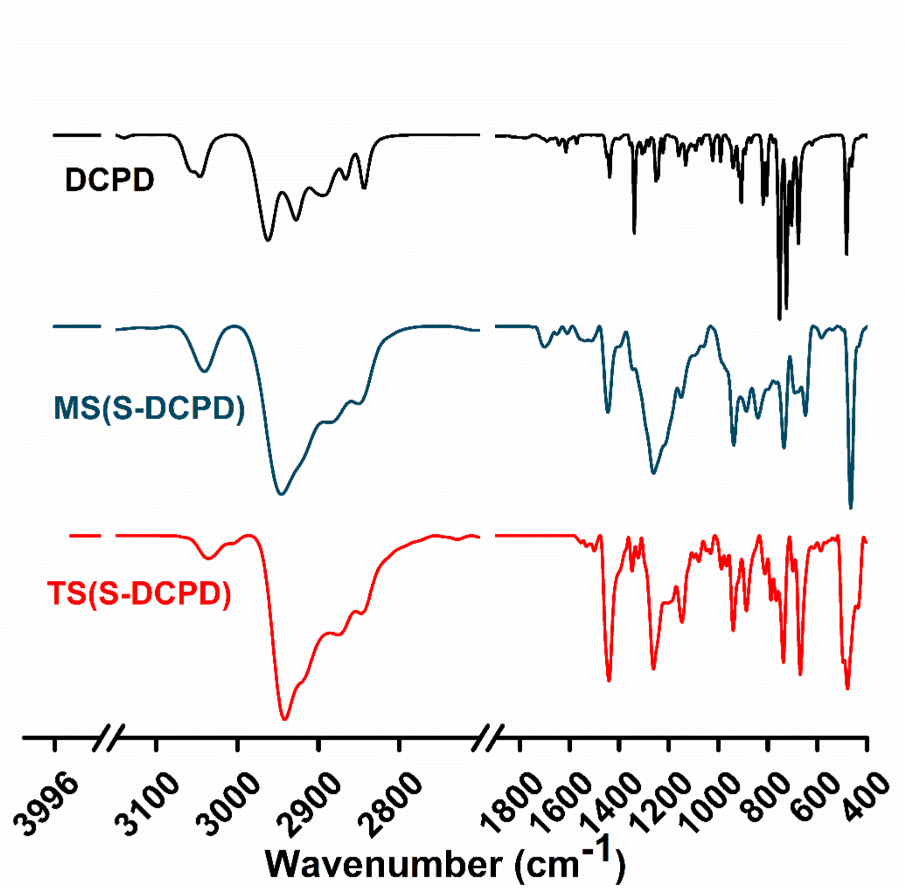

Supplementary Fig. 18. FT-IR curves of monomer DCPD, polymer MS(S-DCPD) and TS(S-DCPD).

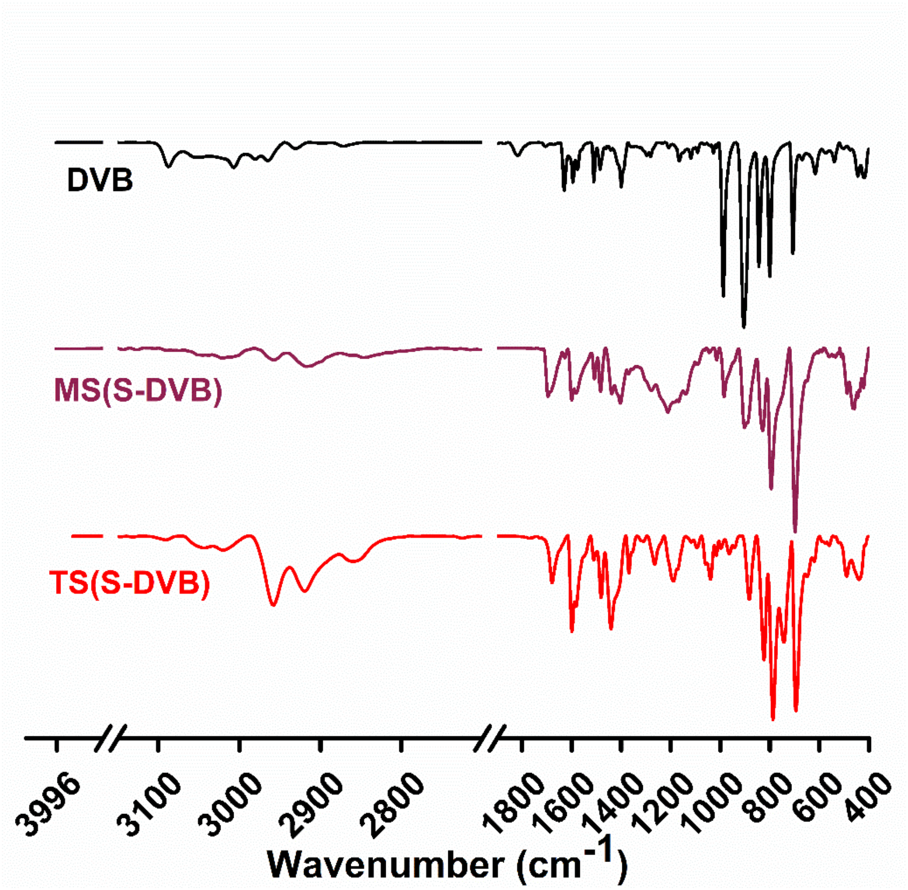

Supplementary Fig. 19. FT-IR curves of monomer DVB, polymer MS(S-DVB) and TS(S-DVB).

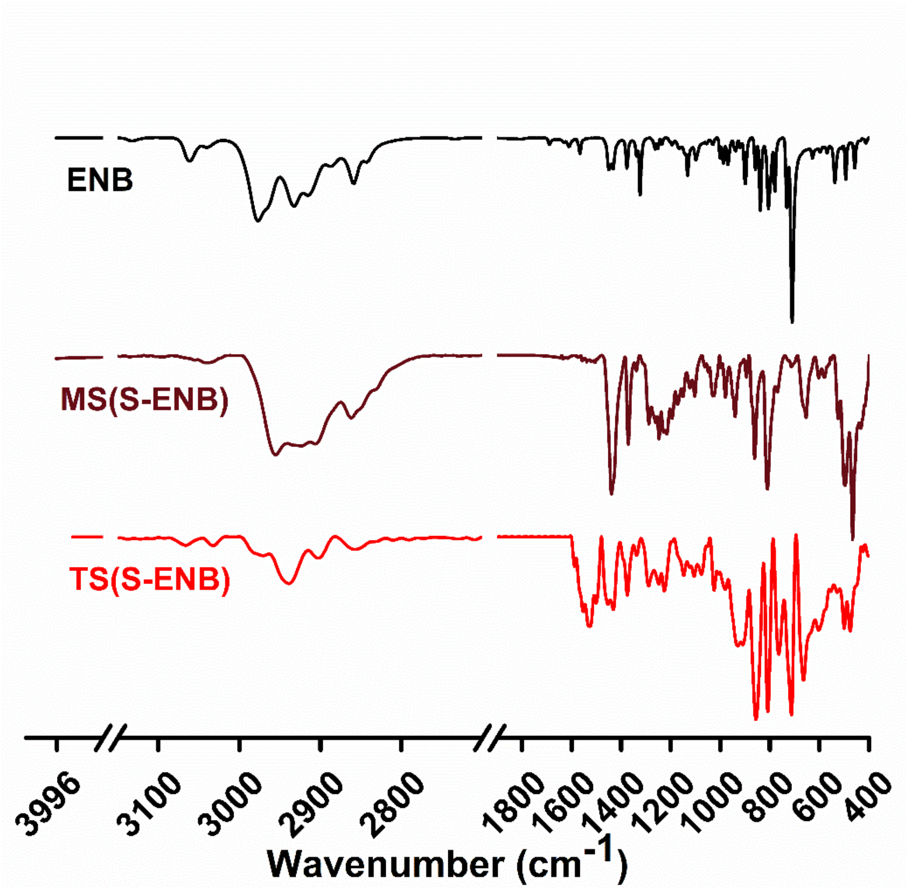

Supplementary Fig. 20. FT-IR curves of monomer ENB, polymer MS(S-ENB) and TS(S-ENB).

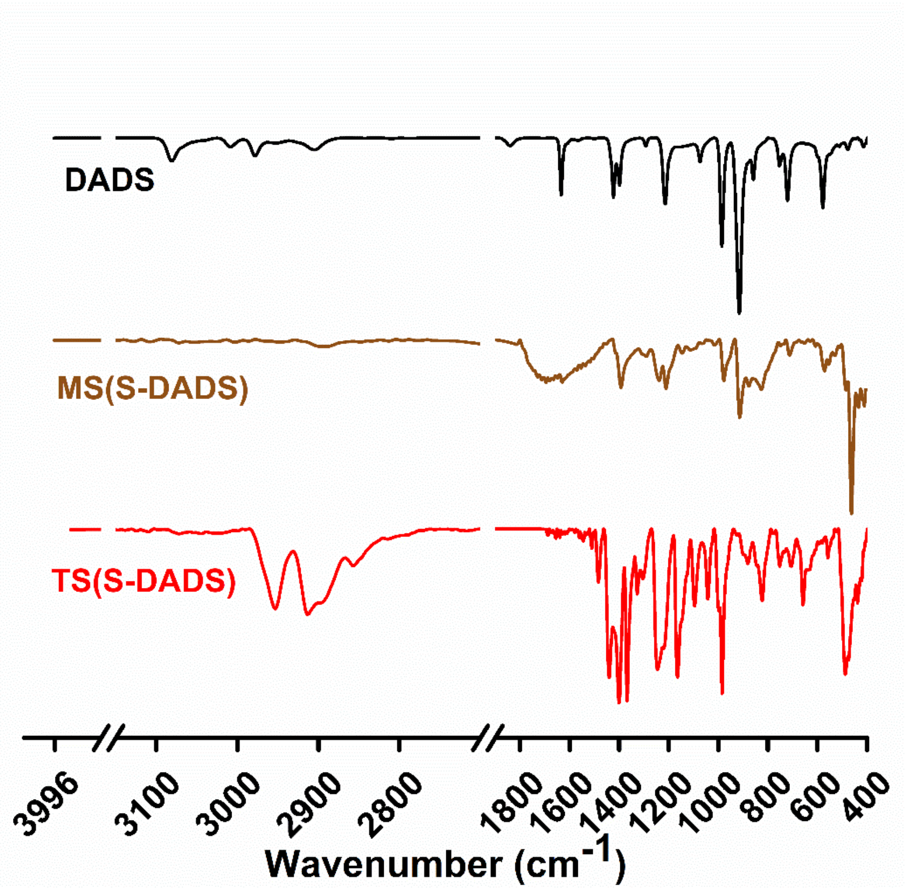

Supplementary Fig. 21. FT-IR curves of monomer DADS, polymer MS(S-DADS) and TS(S-DADS).

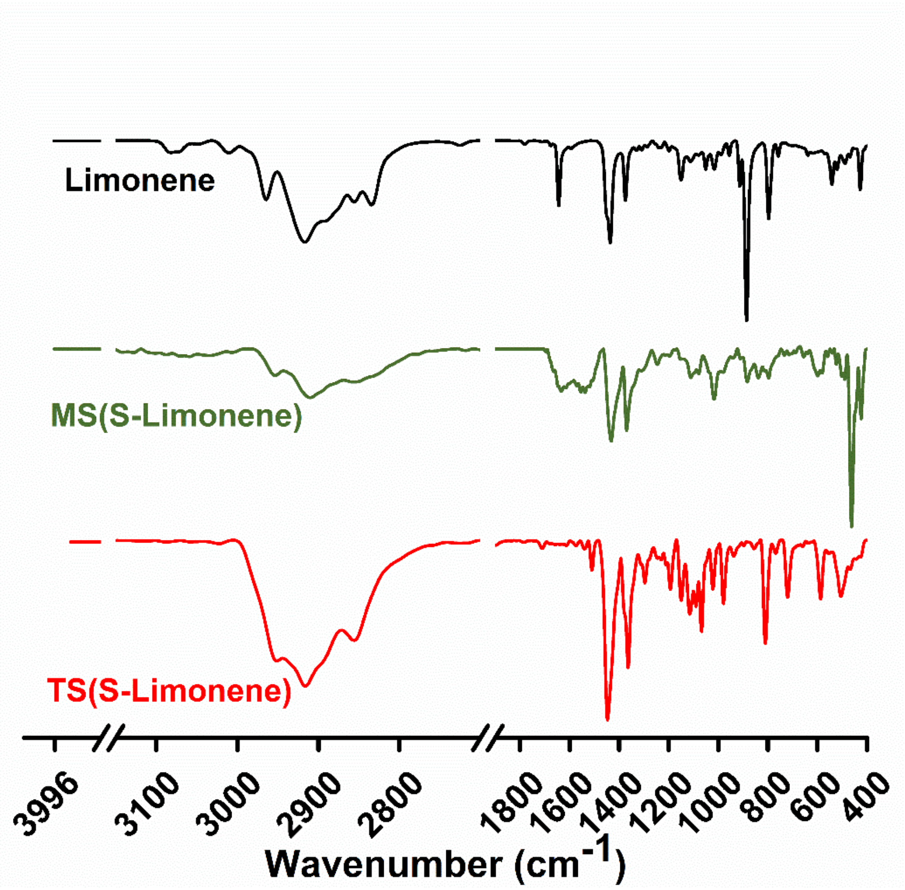

Supplementary Fig. 22. FT-IR curves of monomer limonene, polymer MS(S-Limonene) and TS(S-Limonene).

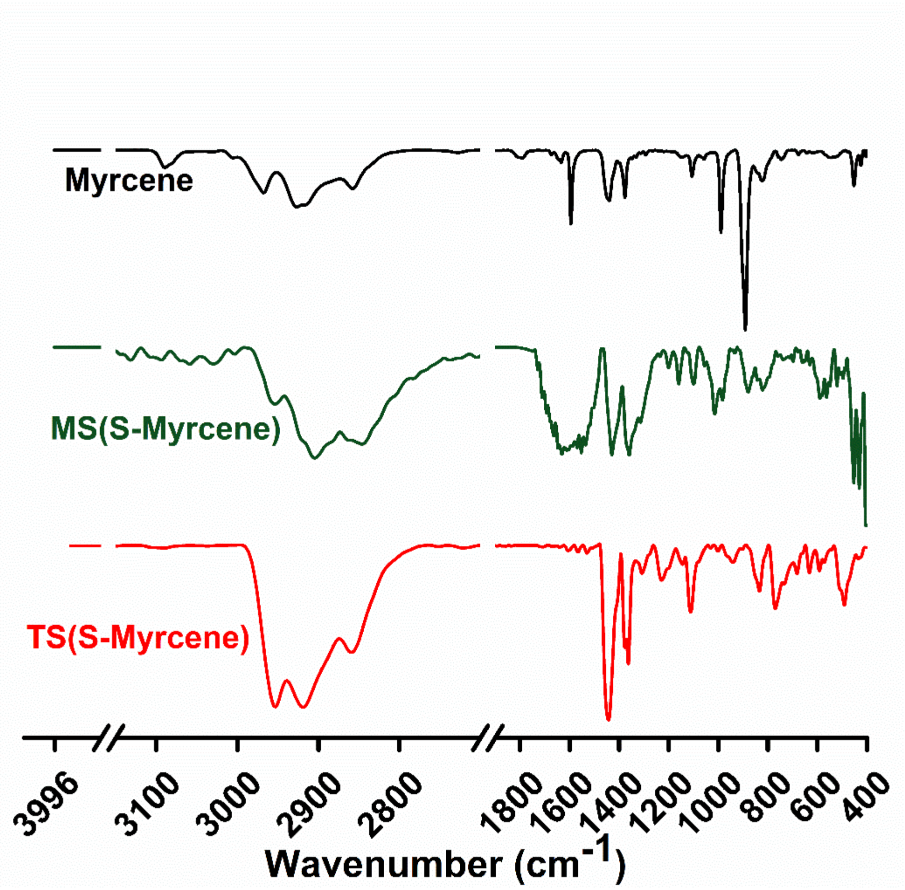

Supplementary Fig. 23. FT-IR curves of monomer myrcene, polymer MS(S-Myrcene) and TS(S-Myrcene).

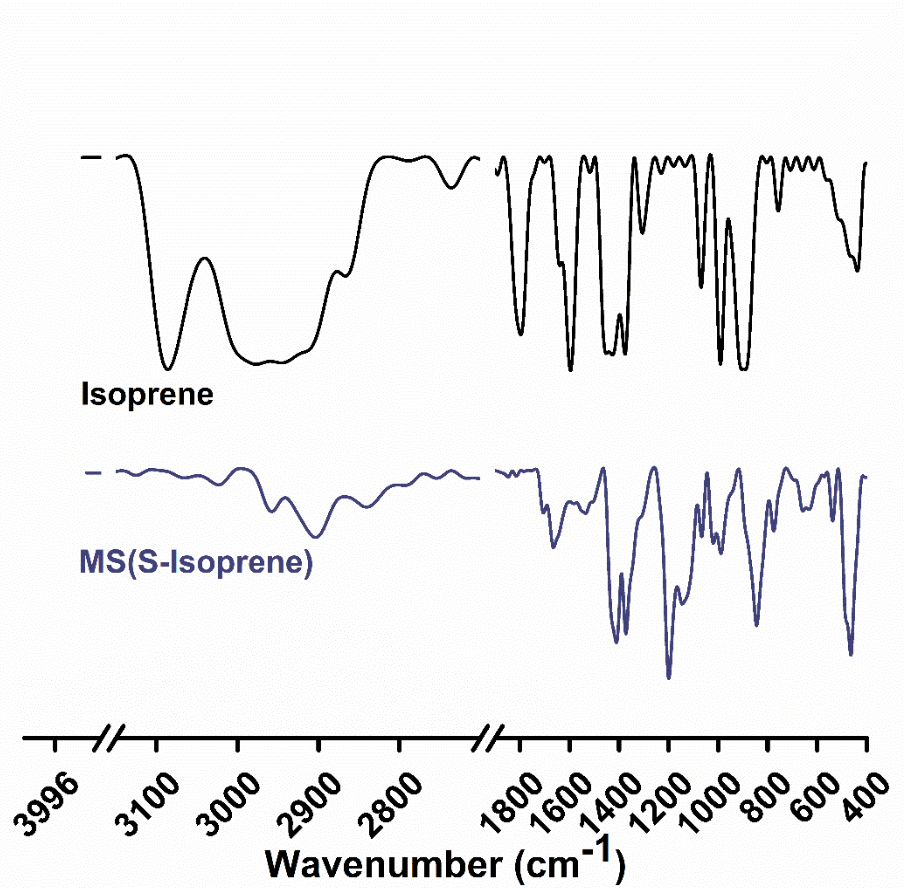

Supplementary Fig. 24. FT-IR curves of monomer isoprene and polymer MS(S-Isoprene).

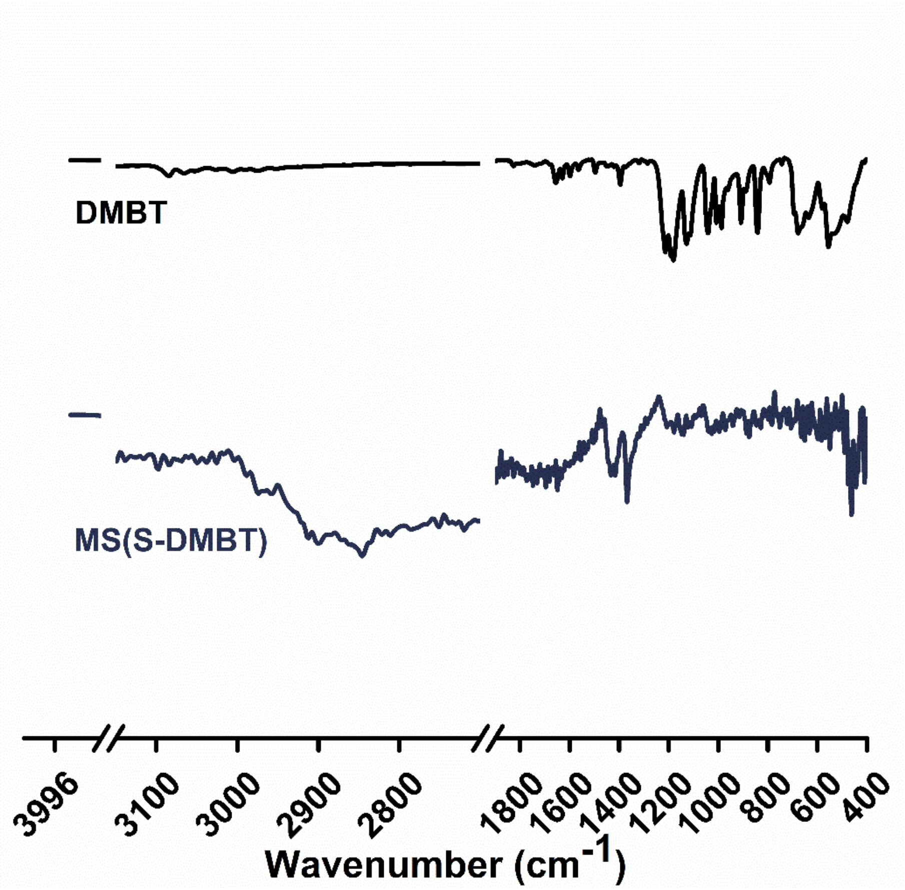

Supplementary Fig. 25. FT-IR curves of monomer DMBT and polymer MS(S-DMBT).

In order to see whether iron is necessary for reaction occurring, ceramic ball mill was used to do synthesis of inverse vulcanized polymers. Monomers DIB and styrene were used in this process. The synthesis procedure was illustrated in experimental section. Reaction time (3 hours) was kept same with that in steel ball milling process. The obtained products were named as MS(S-monomer)_Zirconia. Supplementary Fig. 26 gives the photographs of two products, which shows the resulting products have lighter color than that synthesized using steel ball mill. Moreover, solubility results in Supplementary Fig. 27 show that both products are insoluble in THF and chloroform. Unfortunately, we still cannot get any information using NMR due to insoluble properties of the products. As Figs. S28 and S29 show, what can be detected in the MS(S-Styrene)_Zirconia is totally monomer and no polymer signal can be seen. Other characterizations (DSC, TGA, PXRD and FTIR) were conducted to analyse the properties and structure of products.

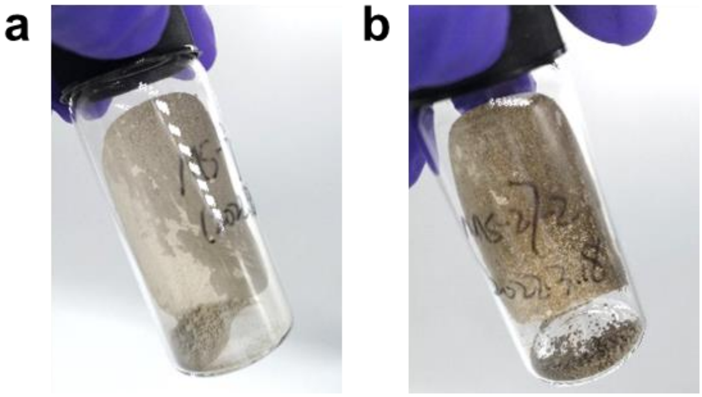

Supplementary Fig. 26. Photographs of products a) MS(S-DIB)_Zirconia and b) MS(S-Styrene)_Zirconia that were synthesized using zirconia ball mill.

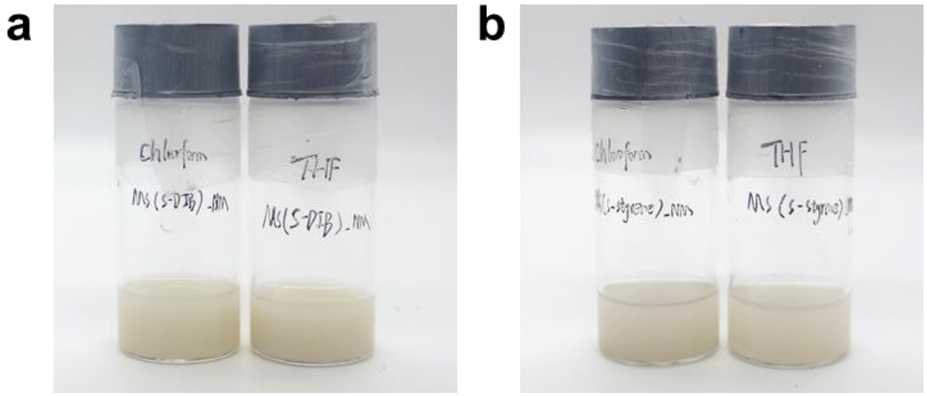

Supplementary Fig. 27. Solubility of a) MS(S-DIB)_Zirconia and b) MS(S-Styrene)_Zirconia in chloroform (left) and THF (right).

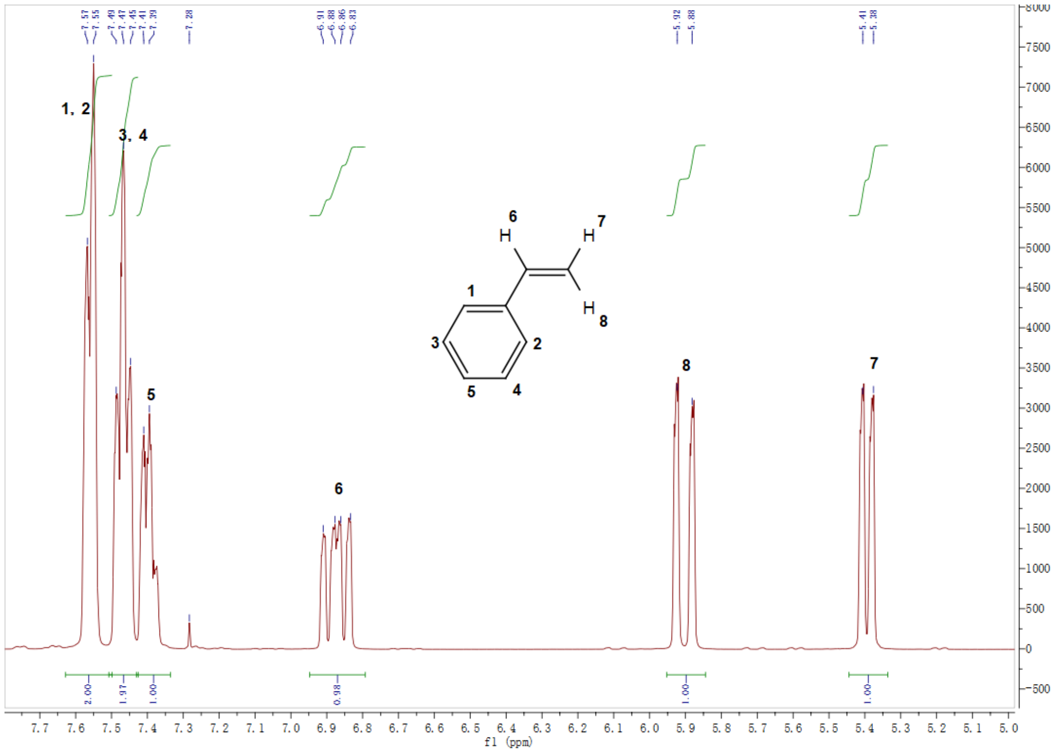

Supplementary Fig. 28. ^{1}H NMR spectra of monomer styrene, conducted at room temperature (~22 ºC). 400 MHz spectrometer and solvent CDCl3 were used.

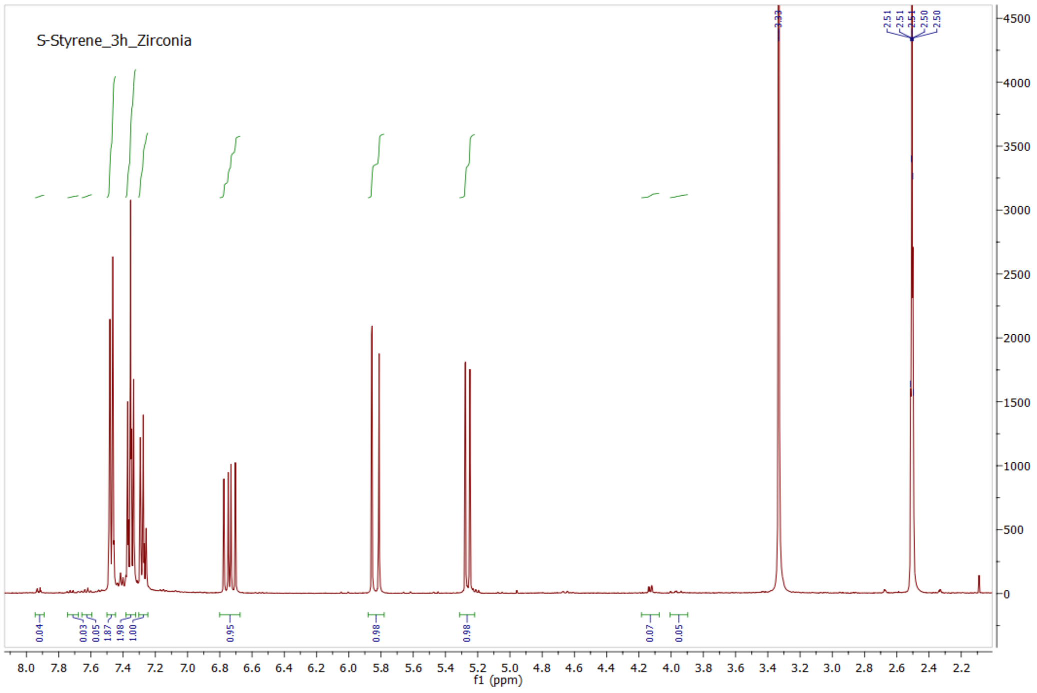

Supplementary Fig. 29. ^{1}H NMR spectra of product MS(S-Styrene)_Zirconia which was directly took out from reaction jar after the reaction finished, conducted at room temperature (~22 ºC). 400 MHz spectrometer and solvent d-DMSO were used.

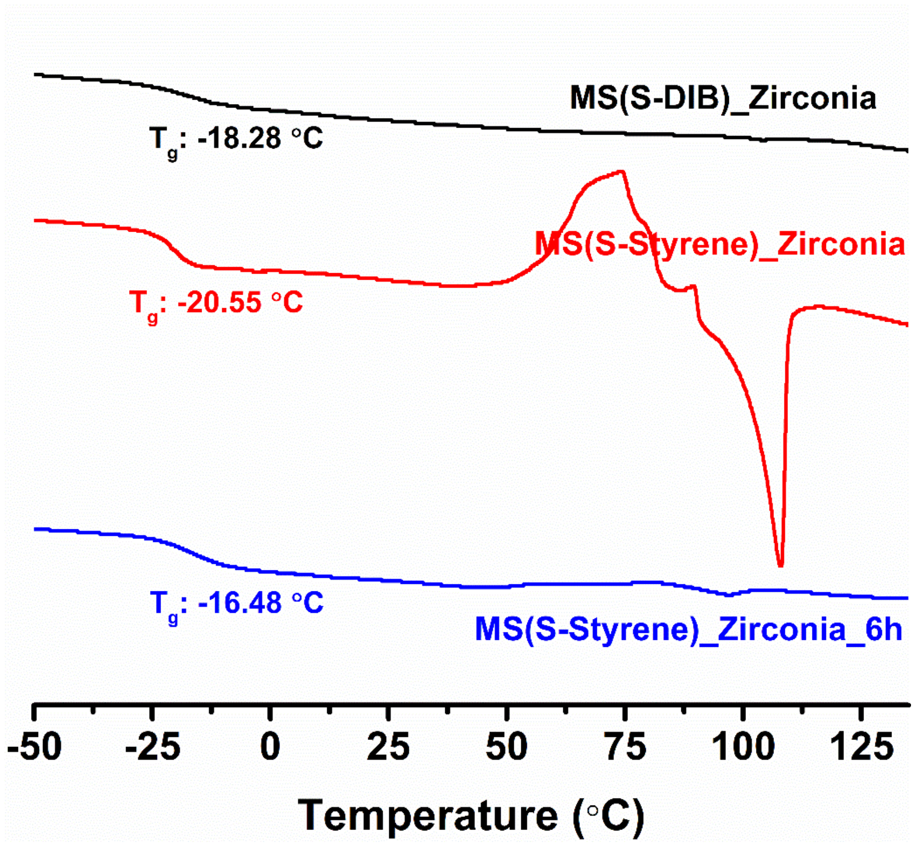

Supplementary Fig. 30. DSC curves of products MS(S-DIB)_Zirconia, MS(S-Styrene)_Zirconia and the product MS(S-Styrene)_Zirconia_6h that was synthesized by extending reaction time to 6 hours.

It is obvious from Supplementary Fig. 30 that MS(S-DIB)_Zirconia has a glass-rubber phase transition at -18.28 °C and there is no unreacted crystalline sulfur in the polymer. That means polymer can be synthesized using non-metal ball mill, and iron is not necessary for this process. Furthermore, we can see that there is unreacted crystalline sulfur in the product MS(S-Styrene)_Zirconia although there is an obvious phase transition occurred at -20.55 °C. However, when the reaction time was extended from 3 hours to 6 hours, there is nearly no unreacted crystalline sulfur remained and the glass transition temperature increased simultaneously. That further suggests that mechanical energy is the key drive force of the reaction rather than the iron. Additionally, MS(S-DIB)_Zirconia has lower T_g than MS(S-DIB)_Steel (in order to distinguish metal and non-metal milled polymer, the polymers that were synthesized using steel ball mill are named as MS(S-monomer)_Steel here), suggesting that higher crosslinking content was obtained using steel ball mill. Two possible reasons we can consider which cause this result. First one is definitely that metal mill generates higher energy than ceramic ball mill when all parameters are consistent (frequency, volume, ball numbers and ball sizes), so that higher reaction degree can be obtained. Another one is that maybe iron acts as a ‘catalyst’ in some content here to accelerate the reaction.

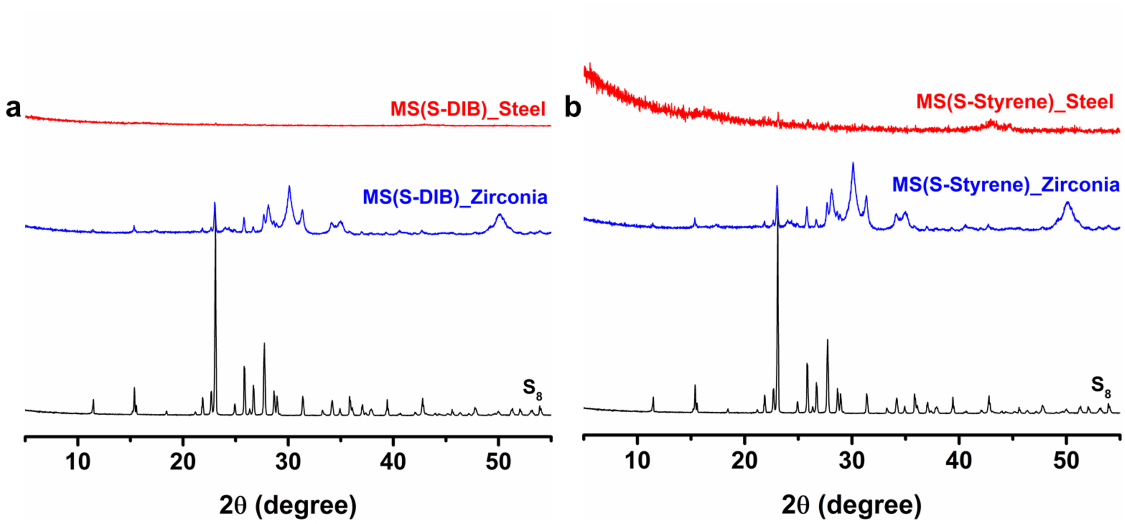

Supplementary Fig. 31. PXRD curves of a) MS(S-DIB)_Zirconia, MS(S-DIB)_Steel and sulfur, and b) MS(S-Styrene)_Zirconia, MS(S-Styrene)_Steel and sulfur.

Figs. S31 shows that there are obvious crystalline materials existing in the both products. The peaks are not from elemental sulfur but is attributed to zirconia.[6] That suggests that debris, metallic or non-metallic, normally falls off the ball during impact, and in this case, zirconia has a similar effect on the polymer as iron does on the polymer, owing to presence of metal element zirconium, i.e. the obtained polymer is insoluble. Additionally, the impurity of the relevant polymers unfortunately results noisy FT-IR signals as shown in Supplementary Fig. 32, and it is hard to make a conclusion on the chemical bonding information.

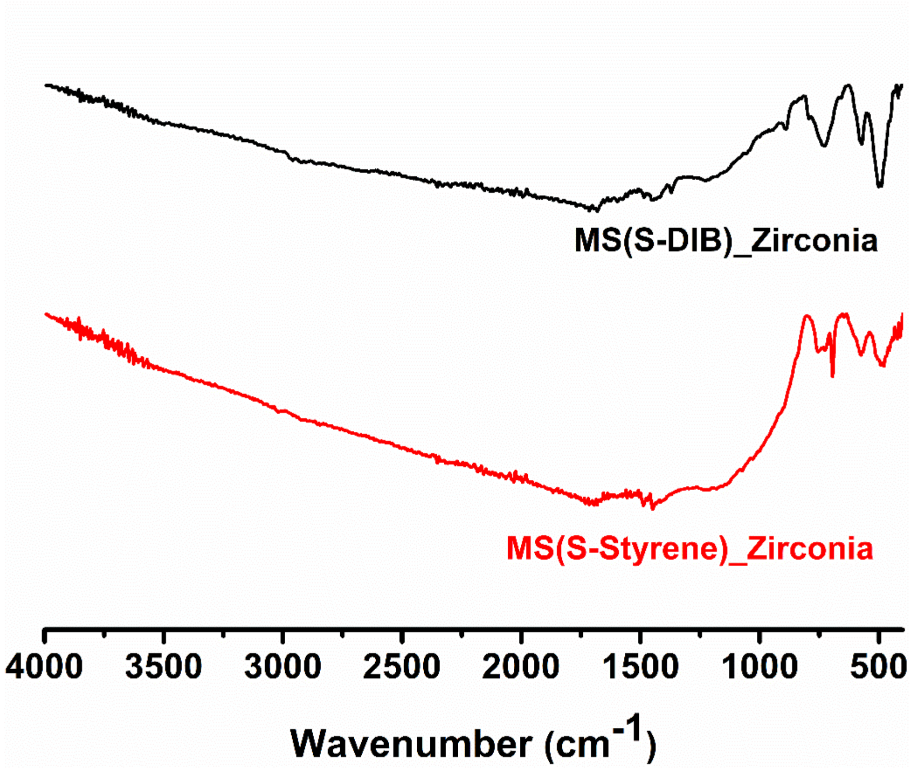

Supplementary Fig. 32. FTIR curves of polymers MS(S-DIB)_Zirconia and MS(S-Styrene)_Zirconia.

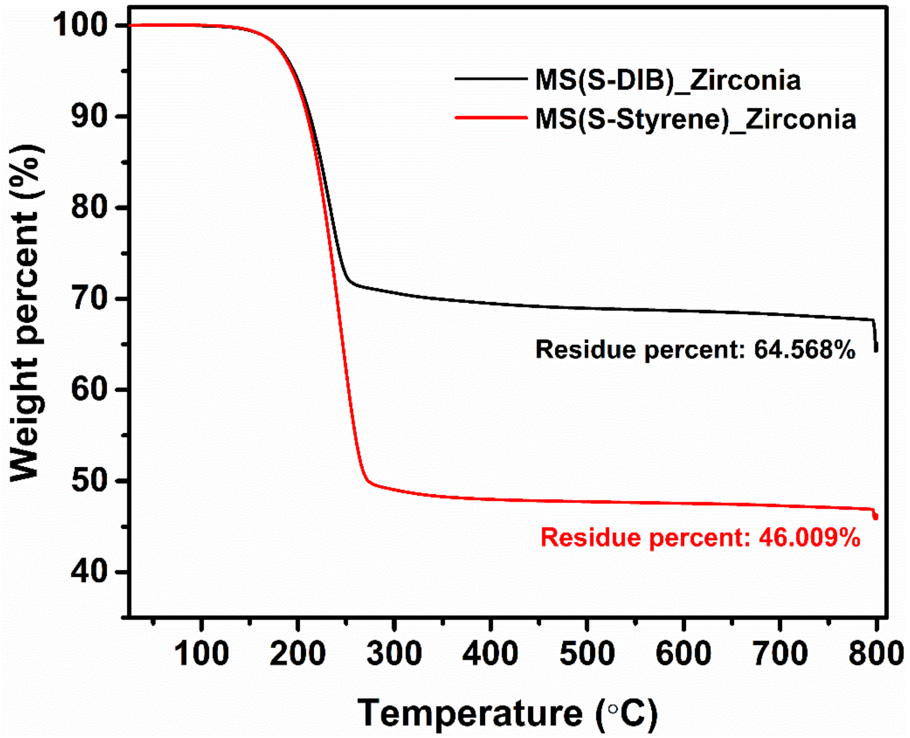

Supplementary Fig. 33. TGA curves of polymers MS(S-DIB)_Zirconia and MS(S-Styrene)_Zirconia, which were run in N_2 ramping from room temperature to 800 °C and were kept at 800 °C for 1 hour in air.

Supplementary Table 5. Comparison of residue percent from elemental analysis results with that from TGA.

	Elemental analysis			Residue percent (elemental analysis)	Residue percent (TGA)
	%C	%H	%S		
MS(S-DIB)_Zirconia	4.84	1.05	32.23	61.88	64.57
MS(S-Styrene)_Zirconia	6.28	1.25	48.26	44.21	41.01

TGA and elemental analysis were conducted to see what percentage of zirconia is in the relevant polymers. The results from elemental analysis is consistent with that from TGA, which both show there are about 60% and 40% zirconia trapped in MS(S-DIB)_ zirconia and MS(S-Styrene)_ zirconia, respectively.

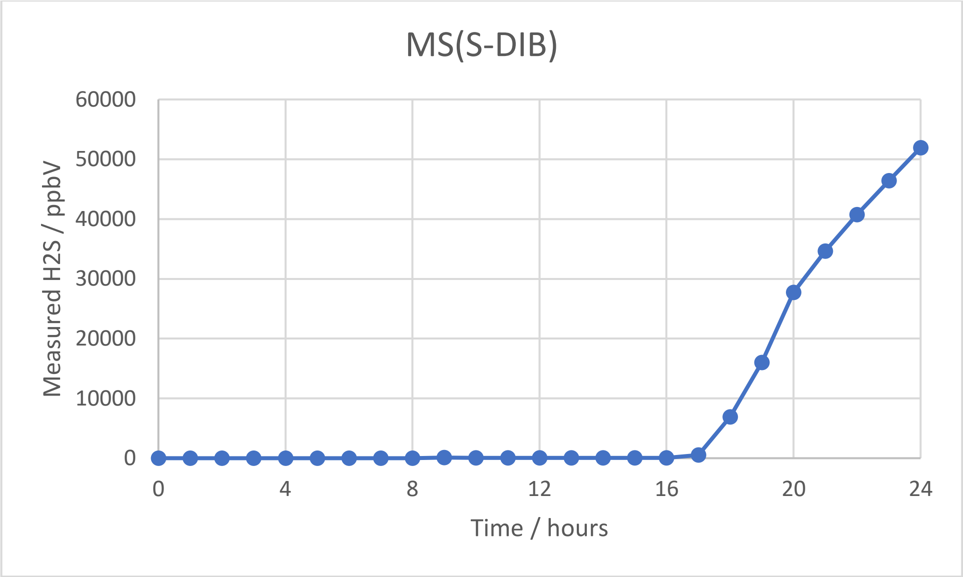

Supplementary Fig. 34. H_2S gas generation measured of the polymer MS(S-DIB) maintained in the air for 24 h with the temperature ramped from room temperature (kept for 8 h) to 80 °C (kept for 8 h) to 140 °C (kept for 8 h).

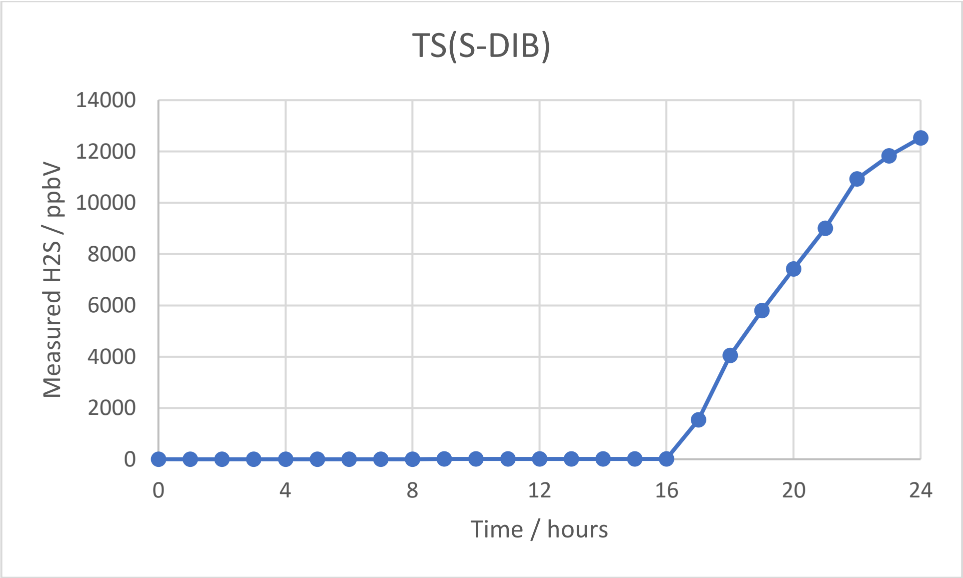

Supplementary Fig. 35. H_2S gas generation measured of the polymer TS(S-DIB) maintained in the air for 24 h with the temperature ramped from room temperature (kept for 8 h) to 80 °C (kept for 8 h) to 140 °C (kept for 8 h).

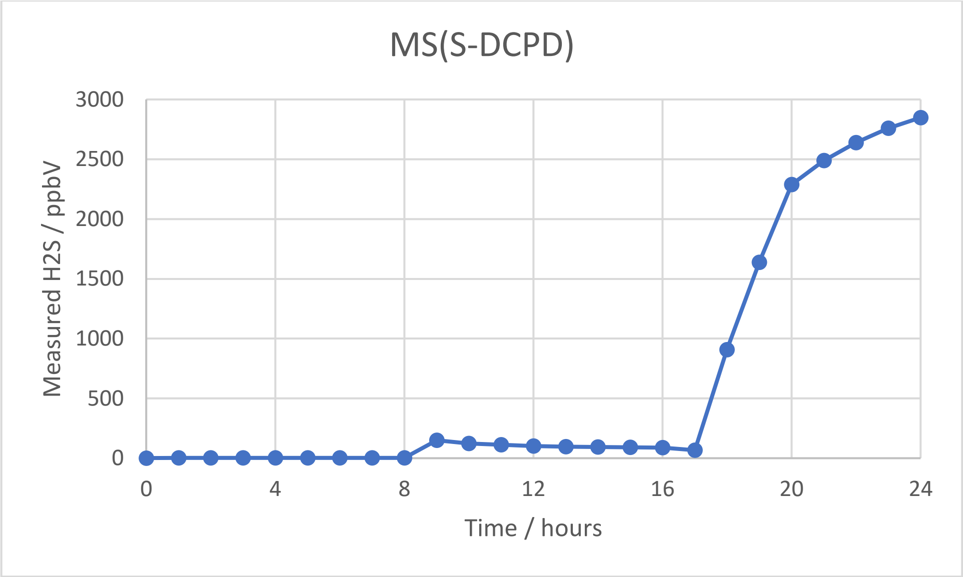

Supplementary Fig. 36. H_2S gas generation measured of the polymer MS(S-DCPD) maintained in the air for 24 h with the temperature ramped from room temperature (kept for 8 h) to 80 °C (kept for 8 h) to 140 °C (kept for 8 h).

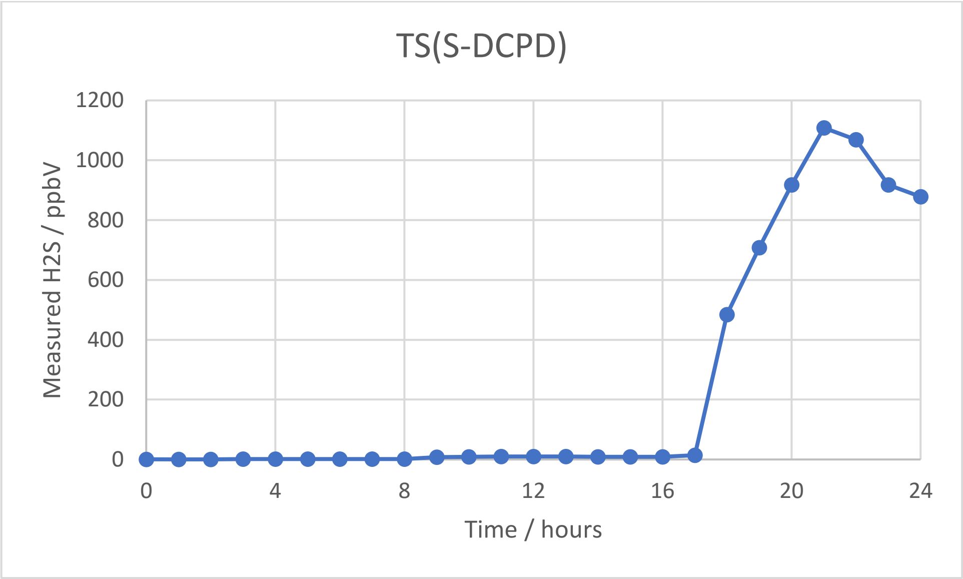

Supplementary Fig. 37. H_2S gas generation measured of the polymer TS(S-DCPD) maintained in the air for 24 h with the temperature ramped from room temperature (kept for 8 h) to 80 °C (kept for 8 h) to 140 °C (kept for 8 h).

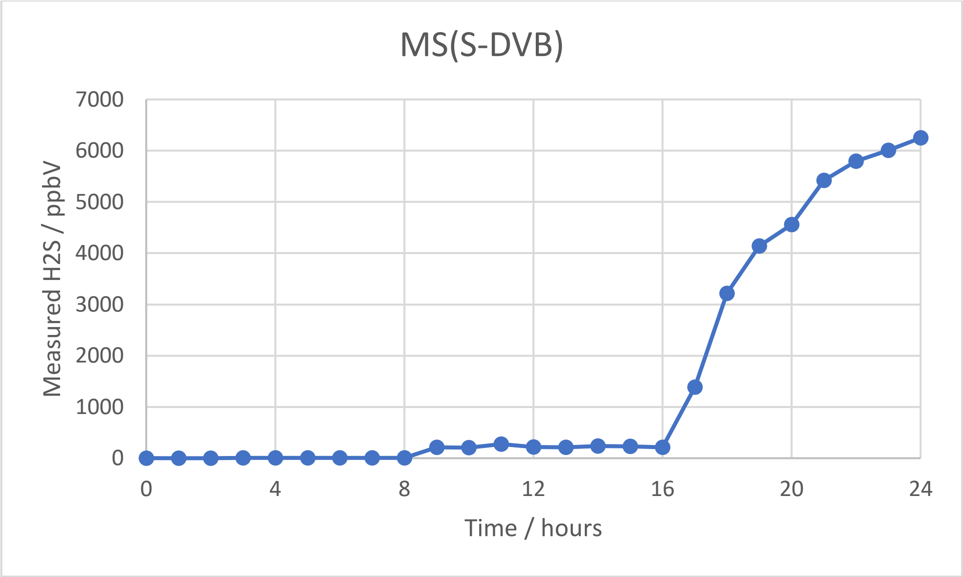

Supplementary Fig. 38. H_2S gas generation measured of the polymer MS(S-DVB) maintained in the air for 24 h with the temperature ramped from room temperature (kept for 8 h) to 80 °C (kept for 8 h) to 140 °C (kept for 8 h).

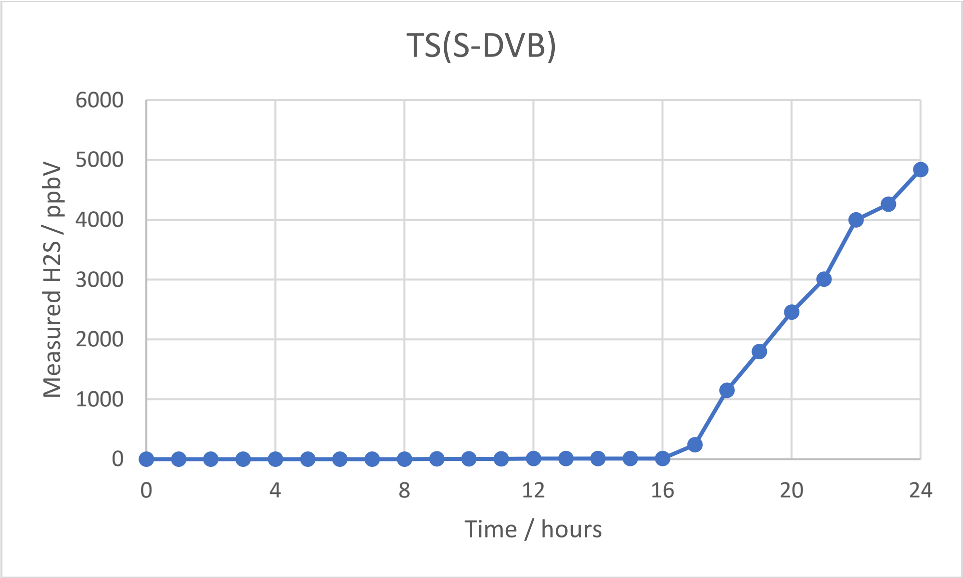

Supplementary Fig. 39. H_2S gas generation measured of the polymer TS(S-DVB) maintained in the air for 24 h with the temperature ramped from room temperature (kept for 8 h) to 80 °C (kept for 8 h) to 140 °C (kept for 8 h).

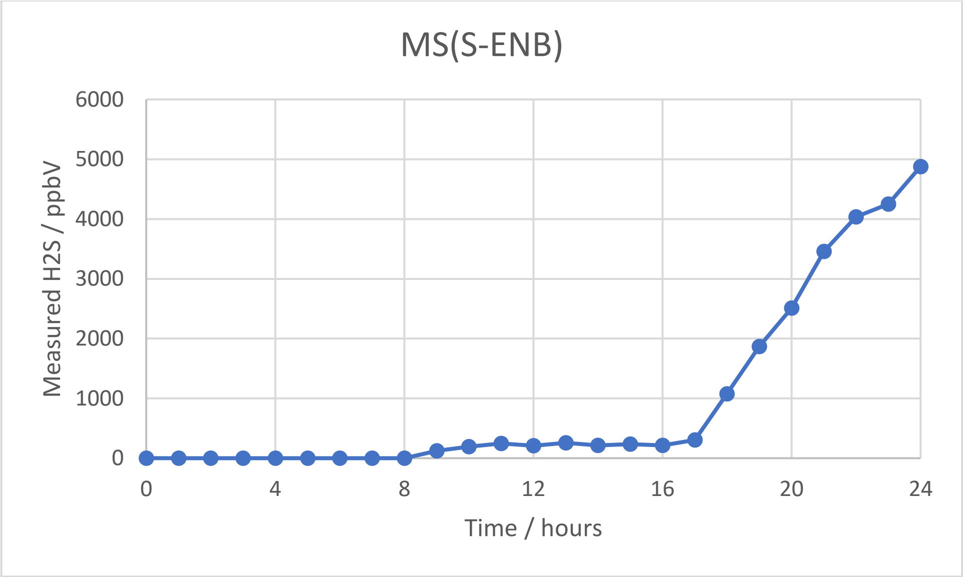

Supplementary Fig. 40. H_2S gas generation measured of the polymer MS(S-ENB) maintained in the air for 24 h with the temperature ramped from room temperature (kept for 8 h) to 80 °C (kept for 8 h) to 140 °C (kept for 8 h).

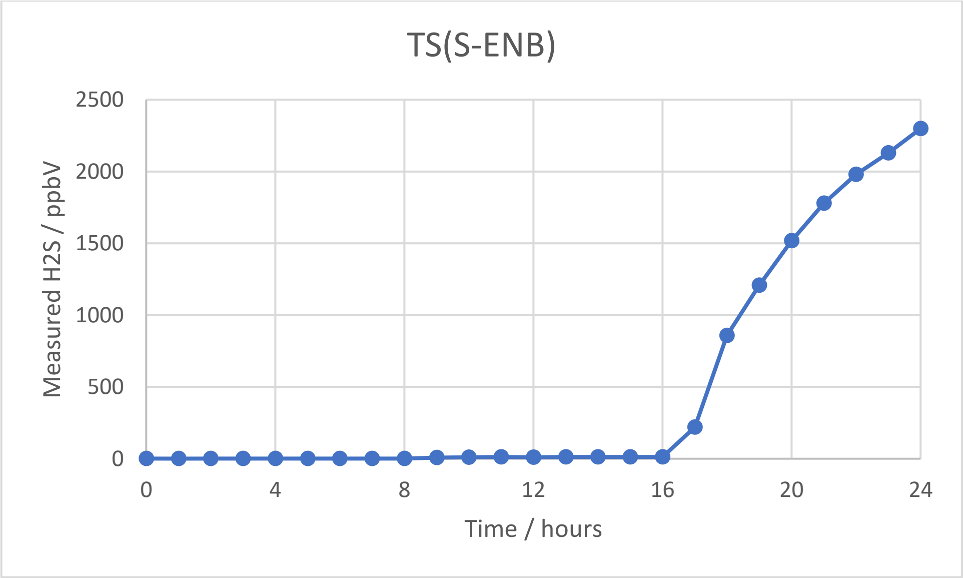

Supplementary Fig. 41. H_2S gas generation measured of the polymer TS(S-ENB) maintained in the air for 24 h with the temperature ramped from room temperature (kept for 8 h) to 80 °C (kept for 8 h) to 140 °C (kept for 8 h).

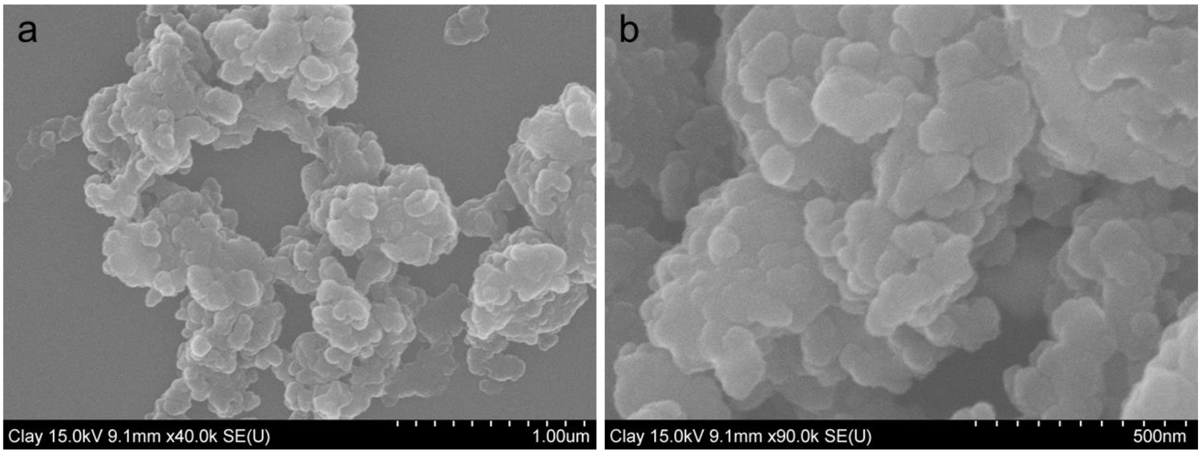

Supplementary Fig. 42. a) SEM images of polymer MS(S-DIB). b) magnification of a section from panel a.

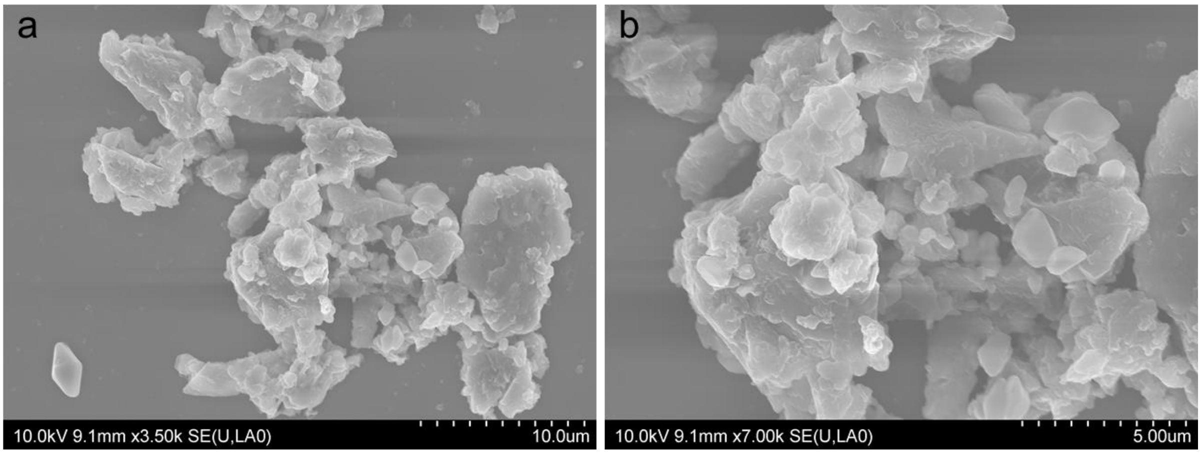

Supplementary Fig. 43. a) SEM images of polymer MS(S-DCPD). b) magnification of a section from panel a.

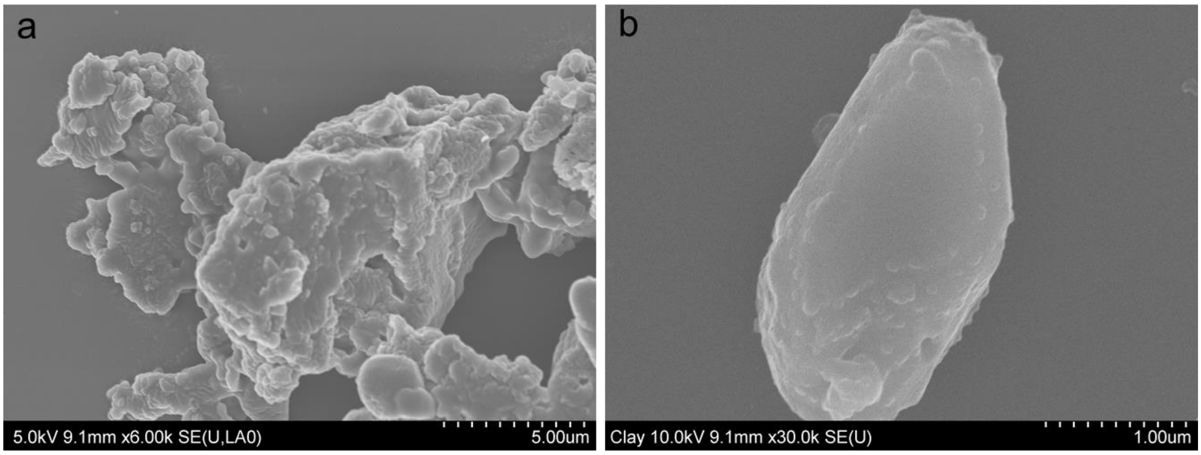

Supplementary Fig. 44. a) SEM images of polymer MS(S-DVB). b) magnification of a section from panel a.

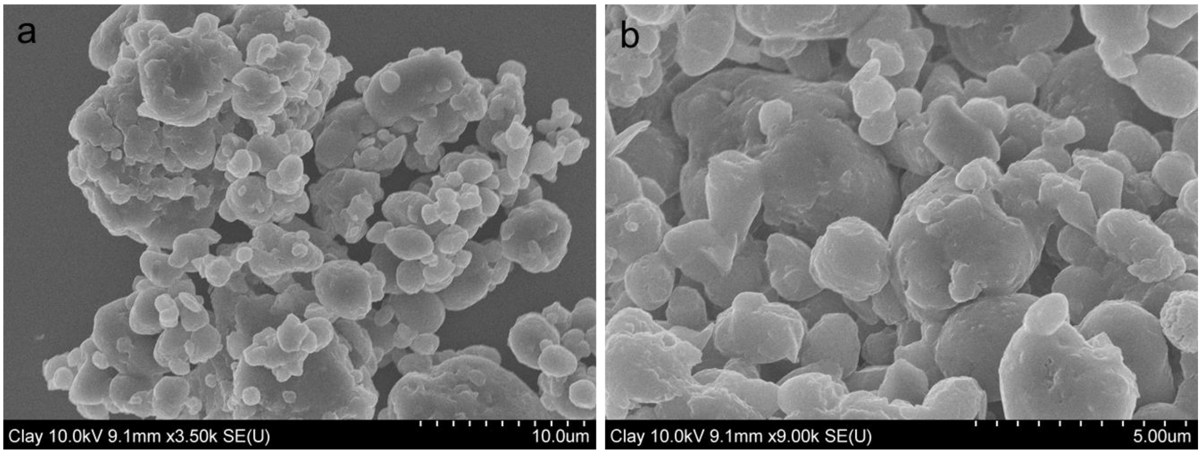

Supplementary Fig. 45. a) SEM images of polymer MS(S-ENB). b) magnification of a section from panel a.

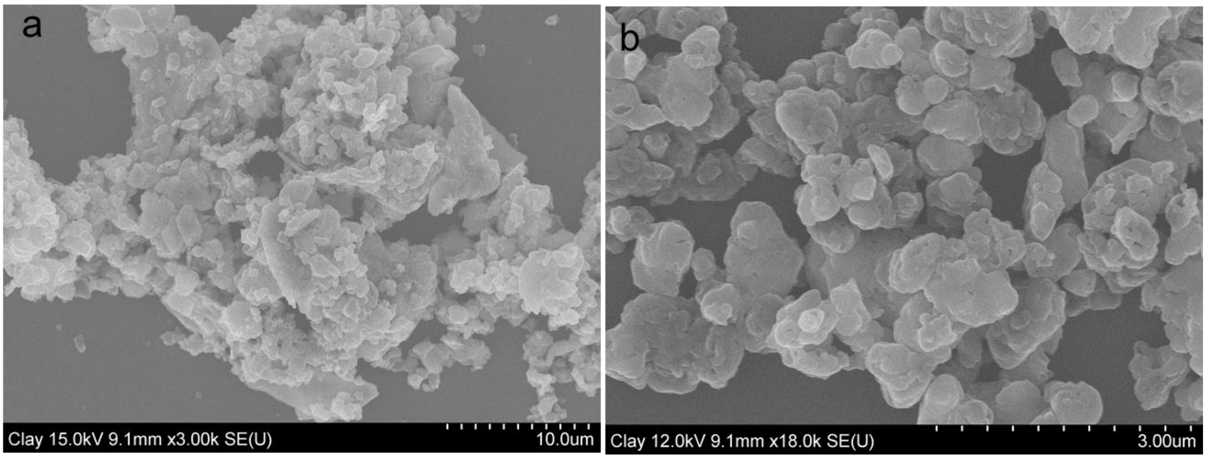

Supplementary Fig. 46. a) SEM images of polymer MS(S-Limonene). b) magnification of a section from panel a.

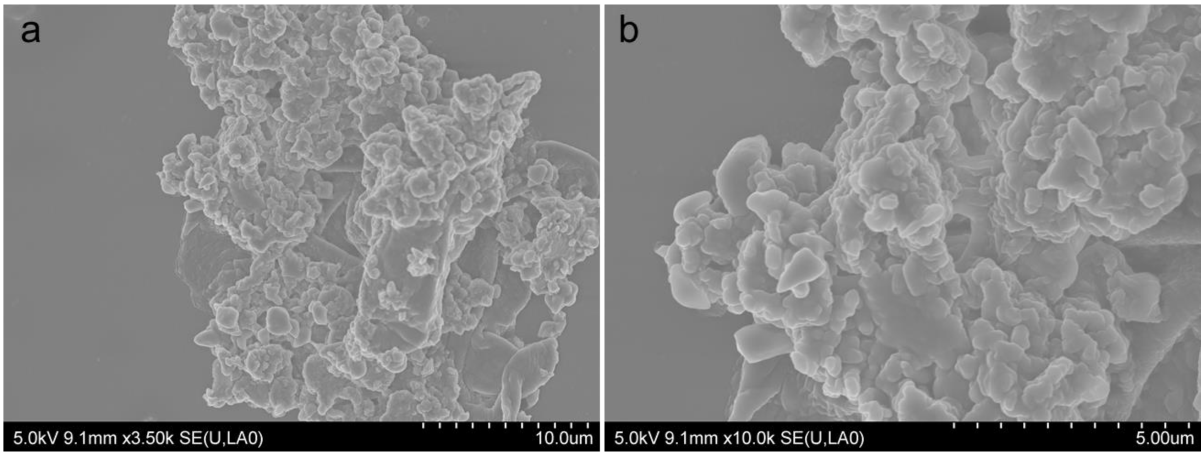

Supplementary Fig. 47. a) SEM images of polymer MS(S-Myrcene). b) magnification of a section from panel a.

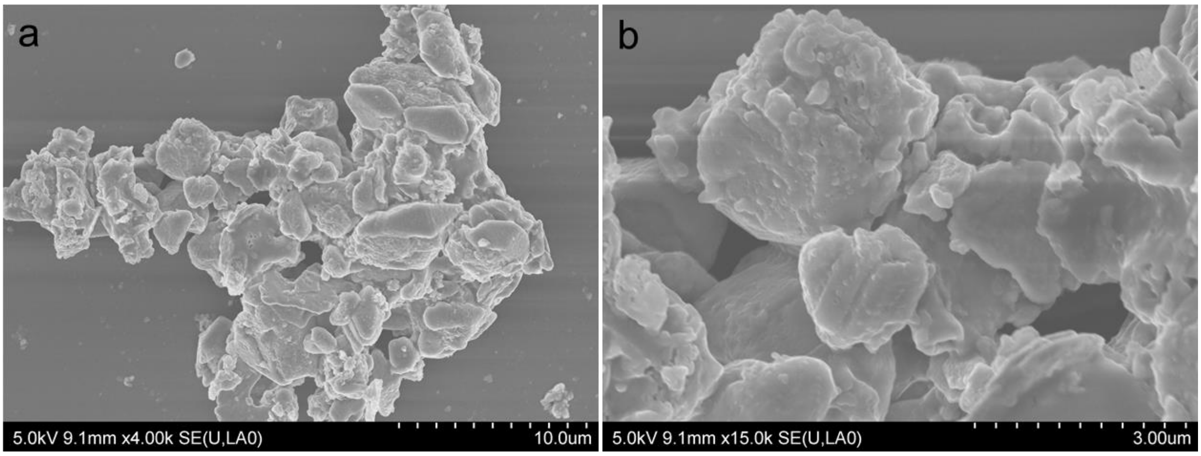

Supplementary Fig. 48. a) SEM images of polymer MS(S-DADS). b) magnification of a section from panel a.

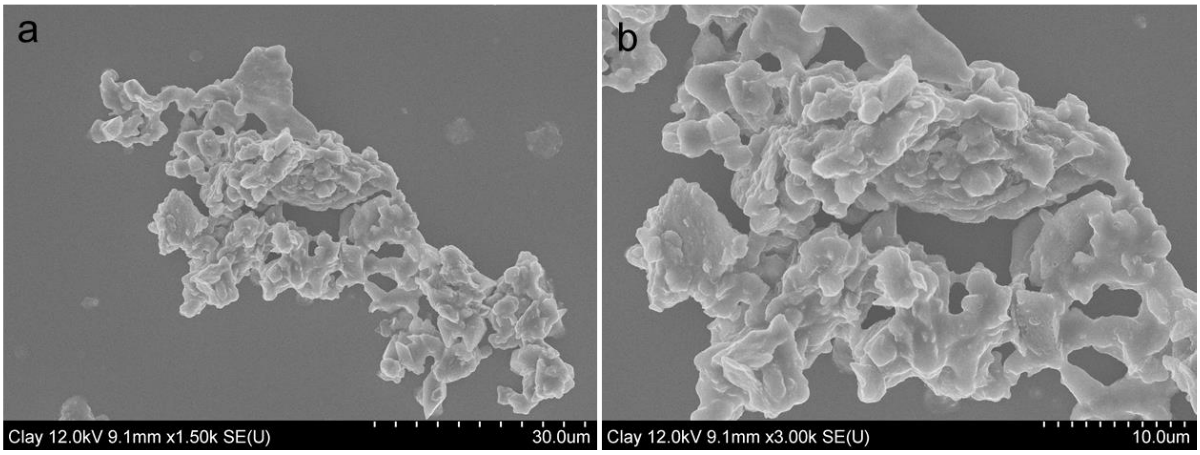

Supplementary Fig. 49. a) SEM images of polymer MS(S-Styrene). b) magnification of a section from panel a.

To compare the mechanical properties of mechanochemically synthesized polymers with that of thermally synthesized polymers, tensile testing of the selected polymers was conducted out. Copolymer MS(S-DIB) and TS(S-DIB) were selected, as other thermally synthesized polymers either too brittle or too sticky, which are not suitable for tensile tests.

However, it was found that fully cured TS(S-DIB) is so brittle that it cannot be used in tensile tests. Hence, partly cured TS(S-DIB) was used here. According to published works from other research teams, the tensile properties of copolymer derived from sulfur and DIB are varying with the change of synthesis procedure. For example, the stress and strain of the copolymer poly(S-r-DIB) with 15 wt.% DIB (poly(S-r-DIB_{15})), carried out by Werner Pauer *et al.*, are 0.85 MPa and 51%, respectively, while the tensile strength of poly(S-r-DIB) with 10 wt.% DIB (poly(S-r-DIB_{10})) is 8.69±0.54 MPa and breaking strain of it is 6.65±2.23 %, reported by Pyun *et al.*.[7]

It needs clear here that the synthesis procedure of the TS(S-DIB), which was used for tensile test in this work, is illustrated as below:

Sulfur (5 g) and DIB (5 g) were added to a 40 mL reaction vial, sealed with a septum, and heated at 160 ℃ with stirring at 900 rpm. The mixture changed from yellow to a dark red viscous mixture after 1 hour and was poured out into a silicone mould. The polymer was placed into the oven to cure at 140 ℃ for 30 min.

Afterwards, the obtained polymer was further processed into testing specimens ready for tensile tests following the procedure illustrated in the characterization and experimental sections. Testing specimens of polymer MS(S-DIB) were prepared as procedure illustrated in the characterization and experimental sections as well.

Tensile properties of MS(S-DIB) and TS(S-DIB) are shown in Figs. S50 and S51 and Supplementary Table 6.

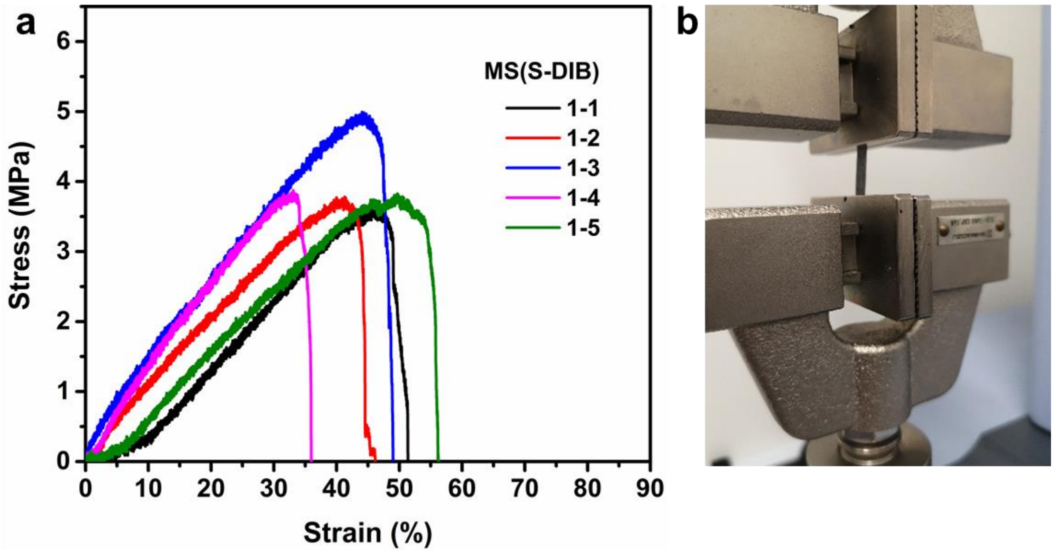

Supplementary Fig. 50. a) Stress-strain curves of polymer MS(S-DIB), and b) photograph of the tensile testing of MS(S-DIB).

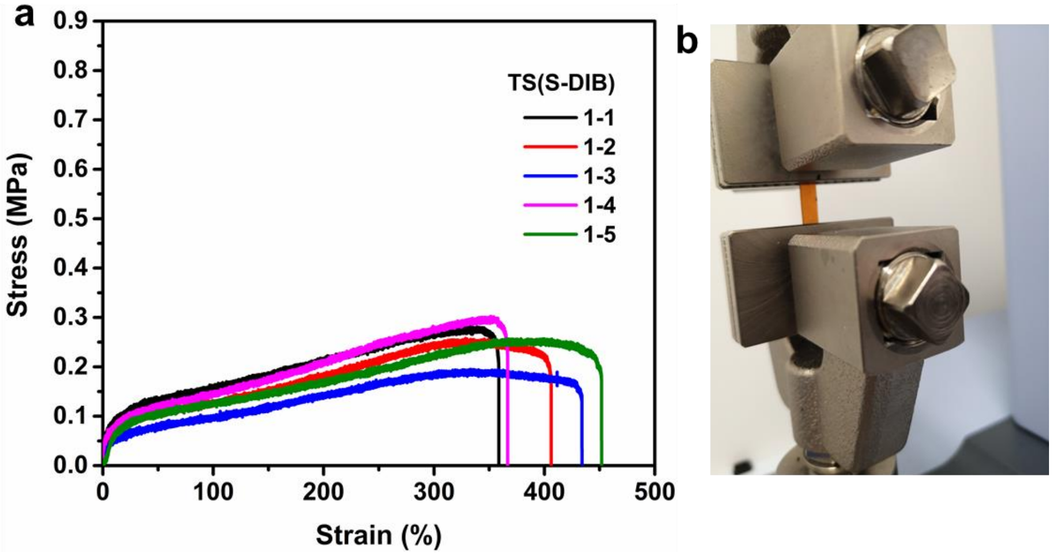

Supplementary Fig. 51. a) Stress-strain curves of polymer TS(S-DIB), and b) photograph of the tensile testing of TS(S-DIB.

Supplementary Table 6. Comparison of tensile properties of MS(S-DIB) with TS(S-DIB).

	Tensile strength (MPa)	Breaking strain (%)	Yong's modulus (MPa)
MS(S-DIB)	3.98±0.33	44.44±8.91	11.37±2.08
TS(S-DIB)	0.26±0.04	349.08±29.63	0.92±0.36

It has been demonstrated that the polymer MS(S-DIB) have higher tensile strength and Yong's modulus than TS(S-DIB), but the values are not comparable to commercial plastics. This material can be used in some applications, which do not have requirements on high mechanical properties of the material, upon its unique properties. Further investigations on the materials with enhanced mechanical properties are needed to explore broaden applications of the materials. That is a worthwhile research direction in the future.

Supplementary References

1. Crockett MP*, et al.* Sulfur‐Limonene Polysulfide: A Material Synthesized Entirely from Industrial By‐Products and Its Use in Removing Toxic Metals from Water and Soil. *Angewandte Chemie International Edition* **55**, 1714-1718 (2016).

2. Khawaja SZ, Kumar SV, Jena KK, Alhassan SM. Flexible sulfur film from inverse vulcanization technique. *Mater Lett* **203**, 58-61 (2017).

3. Parker D*, et al.* Low cost and renewable sulfur-polymers by inverse vulcanisation, and their potential for mercury capture. *J Mater Chem A* **5**, 11682-11692 (2017).

4. Smith JA, Wu X, Berry NG, Hasell T. High sulfur content polymers: the effect of crosslinker structure on inverse vulcanization. *J Polym Sci A: Polym Chem* **56**, 1777-1781 (2018).

5. Zhang Y*, et al.* Inverse vulcanization of elemental sulfur and styrene for polymeric cathodes in Li‐S batteries. *J Polym Sci A: Polym Chem* **55**, 107-116 (2017).

6. Rauta P, Manivasakan P, Rajendran V, Sahu B, Panda B, Mohapatra P. Phase transformation of ZrO2 nanoparticles produced from zircon. *Phase Transitions* **85**, 13-26 (2012).

7. Chung WJ*, et al.* The use of elemental sulfur as an alternative feedstock for polymeric materials. *Nat Chem* **5**, 518-524 (2013).
