# Supplementary material for: Mechanochemical synthesis of inverse vulcanized polymers
Source: Nat Commun. 2022 Aug 16;13:4824. doi: 10.1038/s41467-022-32344-7 (PMC9381570; doi:10.1038/s41467-022-32344-7)
Supplement: Supplementary file 3 — Description of Additional Supplementary Files [file 41467_2022_32344_MOESM3_ESM.pdf]

Title: Supplementary Movie 1

Description: UV-induced self-healing performance
